# Metagenomic comparisons reveal a highly diverse and unique viral community in a seasonally fluctuating hypersaline microbial mat

**DOI:** 10.1099/mgen.0.001063

**Published:** 2023-07-17

**Authors:** Alejandro Miguel Cisneros-Martínez, Luis E. Eguiarte, Valeria Souza

**Affiliations:** ^1^​ Departamento de Ecología Evolutiva, Instituto de Ecología, Universidad Nacional Autónoma de México, Ciudad de México, México; ^2^​ Doctorado en Ciencias Biomédicas, Universidad Nacional Autónoma de México, Ciudad de México, México; ^3^​ Centro de Estudios del Cuaternario de Fuego-Patagonia y Antártica (CEQUA), Punta Arenas, Chile

**Keywords:** Cuatro Cienegas Basin, hypersaline environment, metagenomics, virome, viral diversity

## Abstract

In spring 2016, a shallow hypersaline pond (50×25 m) was found in the Cuatro Cienegas Basin (CCB). This pond, known as Archaean Domes (AD) because of its elastic microbial mats that form dome-shaped structures due to the production of reducing gases reminiscent of the Archaean eon, such as methane and hydrogen sulfide, harbour a highly diverse microbial community, rich in halophilic and methanogenic archaea. AD is a seasonally fluctuating hypersaline site, with salinity ranging from low hypersaline (5.3%) during the wet season to high hypersaline (saturation) during the dry season. To characterize the viral community and to test whether it resembles those of other hypersaline sites (whose diversity is conditioned by salinity), or if it is similar to other CCB sites (with which it shares a common geological history), we generated 12 metagenomes from different seasons and depths over a 4 year period and compared them to 35 metagenomes from varied environments. Haloarchaeaviruses were detected, but were never dominant (average of 15.37 % of the total viral species), and the viral community structure and diversity were not affected by environmental fluctuations. In fact, unlike other viral communities at hypersaline sites, AD remained more diverse than other environments regardless of season. β-Diversity analyses show that AD is closely related to other CCB sites, although it has a unique viral community that forms a cluster of its own. The similarity of two surface samples to the 30 and 50 cm depth samples, as well as the observed increase in diversity at greater depths, supports the hypothesis that the diversity of CCB has evolved as a result of a long time environmental stability of a deep aquifer that functions as a ‘seed bank’ of great microbial diversity that is transported to the surface by sporadic groundwater upwelling events.

## Data Summary

Sequence reads are available on the National Centre for Biotechnology Information (NCBI) Sequence Reads Archive. A full list of accession numbers for metagenomes used in this study are available in File S1, available in the online version of this article. Data processing scripts are publicly available on https://github.com/AleCisMar/COMETS. Supplementary materials are available on Figshare: https://doi.org/10.6084/m9.figshare.20958184[1].

**Impact Statement**

Cuatro Cienegas Basin (CCB) is an endangered oasis in the Chihuahuan Desert of Mexico that, despite its oligotrophic status, is known to harbour great biodiversity, including animals, plants, fungi, and microbes. The antiquity of its sediments, along with the presence of microbial lineages endemic to CCB, adapted to a stoichiometry reminiscent of the Precambrian supereon and related to marine organisms (from which they diverged more than 600 million years ago) and the local abundance of organosedimentary structures (such as microbial mats and stromatolites), have positioned CCB as an analogue of the early Earth and as an Astrobiological park. Archaean Domes (AD) is a unique site within CCB that will deepen our understanding of the origin, diversity and dynamics of microbial communities at this remarkable site. The results presented here show that viral communities at hypersaline sites may be subject to local processes that differentiate them from viral communities at other hyperaline sites around the world, and that viral diversity at different CCB sites may be connected through and nurtured by deep aquifer movements.

## Introduction

Cuatro Cienegas Basin (CCB thereafter) is an endangered oasis in the Chihuahuan desert of Mexico, with N:P ratios in the aquatic environments far from the 16 : 1 Redfield ratio, ranging from very low phosphorus (157 : 1) to very low nitrogen (2 : 1) [2]. Despite its oligotrophic status, CCB is known to harbour a great biodiversity, including animals, plants, fungi and microbes and has been suggested to be an analogue of early Earth for different reasons, including the antiquity of its sediments [3] and the local abundance of organosedimentary structures, such as microbial mats and stromatolites, which are indeed conspicuous in the fossil record dating back to the Archaean eon [4]. The idea that CCB is a model of early Earth is reinforced by the presence of endemic microorganisms, adapted to a stoichiometry reminiscent of the late Precambrian supereon [5], related to marine organisms from which they are estimated to have diverged between 770–680 and 202–160 million years ago [6, 7]. Isotopic studies have shown that CCB aquatic systems are largely composed of aquifer groundwater [8] suggesting that the diversity of the CCB has evolved as a result of long-standing environmental stability of a deep aquifer that recreates ancient ocean conditions [7, 8]. As a result, CCB is considered not only a relevant site for the study of early evolutionary and ecological processes – as illustrated by the discovery of new species and the description of adaptations to extreme environments [7] – but also as an Astrobiological park for the identification of biosignatures that can be used in the search for extraterrestrial life [2].

**Fig. 1.**
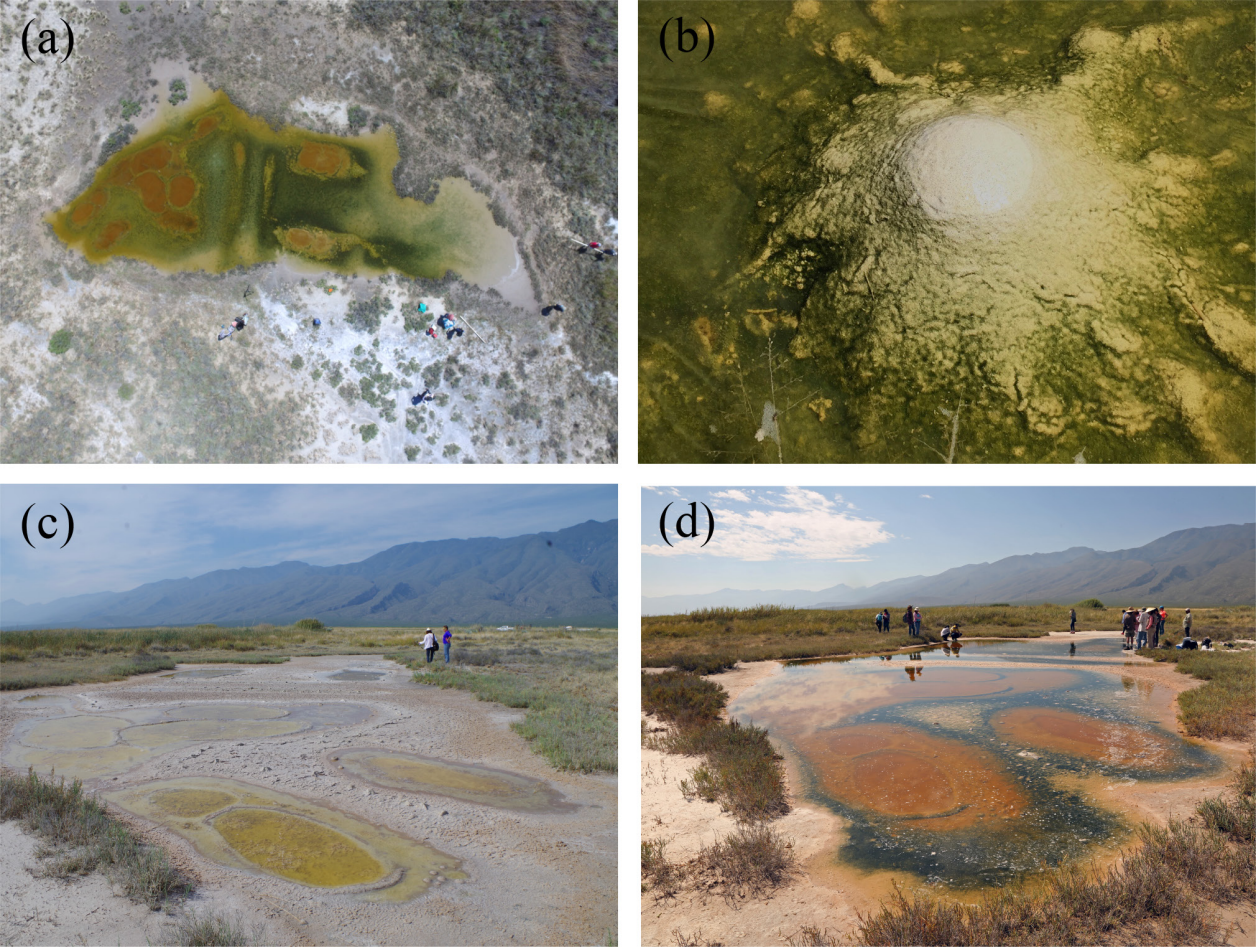
Shallow hypersaline pond, named Archaean Domes or AD from the Cuatro Cienegas Basin (CCB), Mexico. (**a**) Aerial view of the small pond (50×25 m). (**b**) Elastic microbial mat forming a dome shaped structure with a saline crust on top. (**c**) AD during the dry season (April 2016) (**d**) AD during the wet season (September 2019). In C and D Orange Circles or OC are visible.

In spring 2016, a small (50×25 m) and shallow alkaline and hypersaline pond was found in CCB (Fig. 1a). This particular site has ellipsoid cisterns filled with orange water, that we named ‘orange circles’ (OC thereafter) (Fig. 1c, d), that are wet all year and that are particularly rich in clay and silt. Around those OC, in the interphase with sandier sediment, microbial mats form under a salty crust. In that year, after a heavy rain dissolved such salty crust, the microbial mats started to bulge with anoxic gases reminiscent of the Archaean eon, such as methane and hydrogen sulfide [9], forming dome shaped structures (Fig. 1b) and suggesting a deeper connection with the deep aquifer and its deep biosphere. Because of their unique elastic microbial mats and the ‘Archaean like atmosphere’ that was found inside the domes, we called the site “Archaean Domes“ or AD [9, 10]. Given that there are low quantities of Cu metalloenzymes, which is consistent with the low bioavailability of Cu during the Archaean eon [11], and that extreme pH and salinity environments are considered models of ancient Martian ecosystems [12, 13], AD could be considered as a possible model of very ancient Earth communities and a site of astrobiological interest.

AD is a seasonal fluctuating site, with the N:P ratio, pH and salinity ranging from 10 : 1, 9.5 and low hypersaline (5.3 %) during the wet season (Fig. 1d) to 78 : 1, 5.5 and high hypersaline (saturation) during the dry season (Fig. 1c), respectively [9, 10]. Despite its fluctuating and extreme nature, AD seem to harbour a seasonally stable microbial core community, with some degree of functionally redundant taxa [10] and a high abundance of alkaline and salt resistance genes [14]. AD is also very diverse in microbes, including more than 6000 ASV in ten samples obtained at a scale of 1.5 m [10]. This diversity includes a high abundance of halotolerant bacteria, as well as halophilic and methanogenic archaea [9, 10]. AD is particularly rich and diverse in archaea, which are rare in the rest of CCB [9, 10].

Hypersaline environments are characterized by higher salt concentrations than those of seawater (3–4 %) [15] and are usually classified according to their salinity levels; from low salinity (< 10 % NaCl), to intermediate salinity (10 %–20 % NaCl) and high salinity (> 20 % NaCl) [16]. In turn, halophilic organisms are classified according to the NaCl concentration that they require for an optimal growth: i) slight halophiles (1–5 %); ii) moderate halophiles (5–20 %), and iii) extreme halophiles (20–30 %) [15]. Some halophilic microorganisms are also high pH-loving organisms or alkaliphiles [17]. Alkaliphiles can thrive in environments with pH >9 [18], however haloalkaliphilic organisms are typically found in saline alkaline lakes, also known as soda lakes. Soda lakes are characterized by highly alkaline water (pH >9) as a result of high carbonate alkalinity (high CO_3_^2-^ and HCO_3_^-^ concentrations) coupled with low Ca^2+^ and Mg^2+^ [19, 20].

Similarly to thalassic hypersaline environments with neutral pH, soda lakes microbial community composition is strongly influenced by salinity, where higher salt concentrations result in highly abundant extreme halophilic archaea belonging to the class *Halobacteria* and thus in a less diverse community [21–23]. However, soda lakes tend to be more diverse than neutral pH hypersaline environments, which is likely related to the high availability of CO_2_ for primary producers [24] and low Ca^2+^ and Mg^2+^ concentrations [23]. Soda lakes are populated by various salinity and pH adapted bacteria and archaea including members of the *Halomonas* group, well represented strains related to *Bacillus alcalophilus* [24] and at higher salt concentrations *Euryarchaeota* of the class *Halobacteria *, the order *Methanosarcinales* [23] and the genera *Natronococcus* and *Natronobacterium* [17, 24] are readily available.

These trends are likely to extend to viruses, where it has been observed that both halophilic archaea and its viruses (haloarchaeaviruses) become more abundant along an increasing salinity gradient, resulting in a decreased microbial diversity [25]. Haloarchaeaviruses belong mainly to the order *Caudovirales* (families *Siphoviridae*, *Myoviridae* and *Podoviridae*) with a smaller proportion belonging to other viral families such as *Sphaerolipoviridae*, *Pleolipoviridae* and *Fuselloviridae* [26].

Viruses, which are known as the most abundant and diverse entities in the world, typically outnumbering bacterial abundance by ten-fold, even in oligotrophic environments [27, 28], are abundant in hypersaline environments (4×10^8^ – 2×10^9^ VLP ml^−1^) [29]. Viruses are key players in microbial communities, where they can increase the genotypic diversity by mediating genetic exchange among bacterial strains, via transduction, and can also influence host community diversity by selectively killing the densest and most abundant population of fast-growing strains (i.e. kill the winner and similar evolutionary scenarios) [27, 30]. A similar fraction of bacterial mortality is attributed to protist grazing and viral lysis (approximately 20%). However, viral lysis may have a greater impact in the microbial community, by increasing the flux of biomass into dissolved organic matter (DOM) and thus decreasing biomass transfer to higher trophic levels (viral shunt) [27, 28]. Recycling of DOM can produce significant changes in the nutrient pool usually stimulating bacterial growth [27]. This may have a greater impact in oligotrophic conditions, where biomass consists primarily of microbes, and on hypersaline environments, where protist grazing has been seen to disappear at salinities greater than 20 % [31], and therefore haloarchaeaviruses are expected to play the most important role in controlling the halophilic microbial communities.

Different metagenomic studies in CCB have described the viral community within stromatolites from the Río Mezquites river, thrombolites from Pozas Azules II pond [32] and water samples from Churince, La Becerra and Pozas Rojas ponds [33]. These communities are typically dominated by dsDNA bacteriophages from the *Caudovirales* families *Siphoviridae*, *Myoviridae* and *Podoviridae*, followed by ssDNA bacteriophages from the family *Microviridae*, a variety of DNA and RNA viruses from different eukaryotic viral families and virtually no virus infecting archaea [33]. Together, these studies show that the CCB viral communities tend to reflect the diversity patterns of their hosts, displaying a high diversity within and between sites, taxonomic similarity to marine samples and strong signals of endemism [32, 33].

Although viruses are expected to play a significant role in AD microbial dynamics, its viral fraction has not been explored yet. We believe that AD is a microbial community similar to the ones observed in Buck Reef Chert 3.4 Gy fossils in South Africa [34], and that the study of the viruses at AD and their dynamics in time and space will help us to understand the early diversification of life. Given the high salinity of AD, we expected to find a viral community unlike any other within CCB, similar those in other hypersaline environments dominated by haloarchaeaviruses of the order *Caudovirales* [26], and a distribution of diversity in accordance with the reported global patterns of hypersaline viral communities that are driven by salinity levels [25]. Therefore, given the fluctuating conditions of AD, we expected to find clear differences in the community structure between wet and dry seasons. Alternatively, we expected to find a viral community similar to those of other CCB sites due to the influence of groundwater movement produced by the magmatic pouch in the depths of Sierra San Marcos y Pinos, which is known to be a major source of water for aquatic systems in CCB [8].

Here we describe the structure and diversity of the viral fraction within the AD microbial community by analysing twelve metagenomes derived from samples taken from different seasons and depths over a 4 year period. Consistent with the high abundance of archaea in AD, we describe the first occurrence of haloarchaeaviruses in CCB. Although haloarchaeaviruses appear to be an important component of the community at this hypersaline site, the community does not appear to behave as a canonical hypersaline community. More specifically, the community is not dominated by haloarchaeaviruses and its structure is not driven by environmental fluctuations between wet and dry seasons (and thus changes in salinity), which remains highly diverse even at elevated salinity levels (dry season). AD shows the highest viral diversity compared to other sites in CCB and other reported viromes of the world. We found that despite showing some similarities to other hypersaline viral communities (i.e. the presence of haloarchaeaviruses), the viral community in AD is more similar to other CCB viral communities where it shares a common geological history. The similarity in the taxonomic profiles of metagenomes derived from surface samples from 2019 and 2020 with those of metagenomes derived from samples taken at 30 and 50 cm depth (which tend to be more diverse and show higher haloarchaeavirus abundance) suggests that viral diversity may be affected by the sporadic upwelling of groundwater carrying diverse microorganisms from the depths.

## Methods

### Sample collection

Samples were collected inside Rancho Pozas Azules (26°49'41.9"N 102°01'23.6"W) which belongs to Pronatura Noreste, in the Cuatro Ciénegas Basin (CCB), in Coahuila, Mexico (Fig. 1), under SEMARNAT scientific permit number SGPA/DGVS/03121/15.

Samples were collected in April 2016, October 2016, February 2017, October 2018, March 2019, September 2019, and October 2020. Samples taken between February and April correspond to the dry season, while the samples taken between September and October correspond to the wet season. For microbial mats, surface samples were collected by means of a sterile scalpel dissection (8 cm^2^ / 40 cm^3^) and transferred to 50 ml conical tubes. For deeper samples, 30 cm plastic tubes were used as sediment samplers at depths of 30 and 50 cm. Three samples were collected at the shallow ellipsoid orange pools or orange circles (OC): one superficial water sample on a 50 ml conical tube and two more at depths of 30 and 50 cm, as previously described.

In total 12 samples were taken: four microbial mat superficial samples during the dry seasons (M1, M3, M5; D0); three microbial mat superficial samples during the wet seasons (M2, M4, M6); one superficial water sample at OC during a dry season (C0); two microbial mats deep samples during a wet season (D30, D50) and two OC deep samples during a wet season (C30, C50). All samples were stored in liquid nitrogen until processing.

### DNA extraction and sequencing

DNA was extracted according to [35] at the Laboratorio de Evolución Molecular y Experimental of the Instituto de Ecología, Universidad Nacional Autónoma de México, in Mexico City. Briefly, the extractions followed a column based protocol with a Fast DNA Spin Kit for Soil (MP Biomedical). Total DNA was sent to CINVESTAV-LANGEBIO, Irapuato, México for shotgun sequencing with Illumina Mi-Seq paired-end 2×300 technology. The number of raw reads produced for every sequencing is shown in Table 1.

**Table 1.** AD sample and metagenome features. Superficial microbial mat samples taken between April 2016 and September 2019 during dry (M1, M3 and M5) and wet (M2, M4 and M6) seasons, respectively, were used to analyse seasonal variations. In 2020 three microbial mat samples (D0, D30 and D50) and three orange circle samples (C0, C30 and C50) were taken at 0, 30 and 50 cm depth, respectively, to have an approximation on the possible influence of groundwater upwelling on viral diversity. The number of filtered reads varies from 4 412 620 in M2 to 26 799 269 in M1

Sample name	Sample type	Sampling date	Season	Raw reads	Filtered reads	Unclassified %	Bacteria %	Archaea %	Eukaryota %	Viruses %
M1	Microbial mat	April 2016	Dry	28 859 454	26 799 269	40.70	56.36	1.89	0.69	0.35
M2	Microbial mat	October 2016	Wet	4 772 053	4 412 620	41.67	56.09	1.26	0.72	0.25
M3	Microbial mat	February 2017	Dry	8 203 484	7 484 431	37.26	61.03	0.76	0.76	0.19
M4	Microbial mat	October 2018	Wet	10 030 782	9 442 166	39.23	58.26	1.58	0.69	0.24
M5	Microbial mat	March 2019	Dry	25 873 990	24 402 939	38.64	44.12	16.11	0.66	0.47
M6	Microbial mat	September 2019	Wet	20 153 088	19 486 258	37.97	44.71	16.21	0.63	0.48
D0	Microbial mat	October 2020	Wet	17 148 993	15 895 120	42.81	50.98	4.85	0.68	0.68
D30	Microbial mat	October 2020	Wet	18 976 795	18 350 997	52.22	40.78	5.83	0.82	0.35
D50	Microbial mat	October 2020	Wet	16 106 607	15 418 160	51.54	43.19	4.16	0.80	0.30
C0	Water	October 2020	Wet	24 065 589	22 124 414	47.25	44.34	7.16	0.56	0.69
C30	Sediment	October 2020	Wet	14 315 374	13 901 353	53.33	28.84	16.88	0.55	0.40
C50	Sediment	October 2020	Wet	18 050 094	17 604 941	55.42	34.74	8.60	0.71	0.54

### Metagenomic data download

Thirty-five metagenomes available in the literature were downloaded from the Sequence Read Archive (SRA) (File S1, February 2022, https://www.ncbi.nlm.nih.gov/sra), and were grouped into seven types. The metagenomes include 11 previously published CCB viromes [33]; three microbialite viromes [32]; eight high salinity hypersaline viromes [36], one intermediate and one low salinity hypersaline virome [37]; six oceanic viromes [38–40]; and five freshwater viromes [37, 41].

The corresponding SRA accessions are: SRX3861423 (CH2, water from Churince, CCB), SRX3861426 (CH4, water from Churince, CCB), SRX3861413 (CH5, water from Churince, CCB), SRX3861414 (CH9, water from Churince, CCB), SRX3861415 (CH10, water from Churince, CCB), SRX3861416 (BE, water from La Becerra, CCB), SRX3861417 (PR1, water from Pozas Rojas, CCB), SRX3861418 (PR3, water from Pozas Rojas, CCB), SRX3861424 (PR4, water from Pozas Rojas, CCB), SRX3861425 (PR7, water from Pozas Rojas, CCB), SRX3861421 (PR9, water from Pozas Rojas, CCB), SRX000208 (PA, thrombolite in Pozas Azules II, CCB), SRX000209 (RM, stromatolite in Rio Mesquites, CCB), SRX000221 (Highborne cay, stromatolite in Highborne cay, Bahamas), SRX117679 (2007At1, high salinity water from hypersaline Lake Tyrell, Australia), SRX117680 (2007At2, high salinity water from hypersaline Lake Tyrell, Australia), SRX117681 (2009B, high salinity water from hypersaline Lake Tyrell, Australia), SRX117682 (2010Bt1, high salinity water from hypersaline Lake Tyrell, Australia), SRX117683 (2010Bt2, high salinity water from hypersaline Lake Tyrell, Australia), SRX117684 (2010Bt3, high salinity water from hypersaline Lake Tyrell, Australia), SRX117685 (2010Bt4, high salinity water from hypersaline Lake Tyrell, Australia), SRX117686 (2010A, high salinity water from hypersaline Lake Tyrell, Australia), SRX000217 (Saltern low, low salinity saltern in San Diego Bay, USA), SRX000218 (Saltern med, intermediate salinity saltern in San Diego Bay, USA), SRX000215 (Tabuaeran atoll, seawater from Pacific ocean), SRX000213 (Palmyra atoll, seawater from Pacific ocean), SRX000206 (Kiritimati atoll, seawater from Pacific ocean), SRX000204 (Kingman atoll, seawater from Pacific ocean), SRX000202 (Sargasso sea, seawater from Sargasso sea), SRX008299 (Tampa bay, seawater from Tampa bay, USA), SRX000211 (TP_1105, freshwater from Tilapia pond in Kent SeaTech, USA), SRX000235 (TP_0506, freshwater from Tilapia pond in Kent SeaTech, USA), SRX000236 (PP_0506, freshwater from Prebead pond in Kent SeaTech, USA), ERX007894 (Lake Pavin, freshwater from Lake Pavin, France) and ERX007895 (Lake Bourget, freshwater from Lake Bourget, France).

### Metagenomic comparisons

Metagenome comparisons were computed on a custom script (COMETS: COmpare METagenomeS. available in GitHub https://github.com/AleCisMar/COMETS). Briefly, this script takes a metadata table and all the compressed FASTQ files with single- or paired-end raw reads as input. Then, it uses fastp [42] for adapter removal, filtering of low-quality reads (reads with a maximum of 40 % of bases with quality <Q15 are qualified) and deduplication of reads with all identical bases. Next, it uses Kaiju [43] to perform taxonomic classifications by comparing every sequencing read against the nr_euk database. Kaiju uses the Burrows-Wheeler transform to search for matches between sequences of translated reads and the database of microbial coding genes, assigning the taxonomic identifier (from NCBI taxonomy) of the longest exact match to each sequencing read. Kaiju assigns the taxon identifier of the least common ancestor (LCA) if matches of the same length are found in multiple taxa [43]. After the taxonomic classification, COMETS produces a count table and a taxonomy table that are used together with the metadata table to build a phyloseq object [44] in R [45]. In the following step it produces rarefaction curves with the myrlin package [46] and performs a normalization by median sequencing depth.

Finally COMETS generates three types of plots with ggplot2 [47] at different taxonomic levels: taxonomic identifiers assigned by Kaiju are considered OTUs which can be filtered according to taxonomic level, from phylum to species. The first kind of plot is a stacked bar for relative abundance of OTUs that represent at least 1 % of the reads in at least one sample; the second type is a dot plot for alpha diversity (Shannon) and; the last is a non-metric multidimensional scaling (NMDS) scatter plot to represent beta diversity (Bray-Curtis dissimilarity). When taxonomically filtered, OTUs without the corresponding taxonomic assignment (NA) were excluded from diversity calculations and plot generation.

To explore the similarities between viral communities at AD and other viral communities in CCB and the rest of the world, we computed Bray-Curtis dissimilarity measures from the normalized count table at different taxonomic levels to build UPGMA trees with the NEIGHBOR programme from the PHYLYP package, using input order and without subreplicates [48].

### Statistical analyses

For initial statistical analyses, we assumed a normal distribution and performed ANOVA and one-tailed t-Student tests with alpha=0.05 to contrast different groups of samples, i.e. wet (M2, M4 and M6) and dry (M1, M3 and M5) seasons; early surface (M1, M2, M3 and M4), late surface (M5, M6, D0 and C0) and deep samples (D30, D50, C30 and C50) and; surface (M1, M2, M3, M4, D0 and C0) and deep samples (M5, M6, D30, D50, C30 and C50).

For a more robust analysis of the differential abundance between sample groups, we followed the IDEAmex pipeline originally developed for differential expression analysis [49] with the package edgeR [50]. Normalization was performed with the Trimmed Mean of M-values or TMM method, and differentially abundant OTUs were considered with log fold change=1.5 and *p*-value=0.01.

## Results and discussion

### Description of AD microbial community

We analysed the relative abundance of reads assigned to the three domains of life and viruses to explore the general taxonomic structure of each sampled community in AD. As shown in Table 1, a total of 12 samples were analysed: three microbial mat superficial samples during the dry season (M1, M3 and M5) and three microbial mat superficial samples during the wet season (M2, M4 and M6), to evaluate the effect of environmental fluctuations, and three microbial mat samples (D0, D30 and D50) and three OC samples (C0, C30 and C50) at different depths (0, 30 and 50 cm) (see Methods for details), to indirectly evaluate the effect of the deep aquifer on diversity.

The number of filtered reads was on average 16 276 889, ranging from 4 412 620 in M2 to 26 799 269 in M1. On average, most of the reads are assigned to Bacteria (46.95 %), followed by unclassified reads (44.84 %), Archaea (7.11%), Eukaryota (0.69 %) and Viruses (0.41 %). However, these abundances vary over a wide range among the different samples. For example, Bacteria ranged from 61.03 % in the surface dry season sample M3, to 28.84 % in the 30 cm deep OC sample C30, while Archaea varied from 0.76–16.88 % in the same samples (Table 1).

Comparing our samples with the literature, the highest Archaea abundance found in C30 is more similar to the Archaea abundance (16%) reported in Acos, a thalassohaline hypersaline system with intermediate salinity (19 %) in Peru, than the abundance described in Maras, another Peruvian site, which can reach more than 30 % NaCl, where Archaea can reach up to 80–86 % relative abundance [22].

**Fig. 2.**
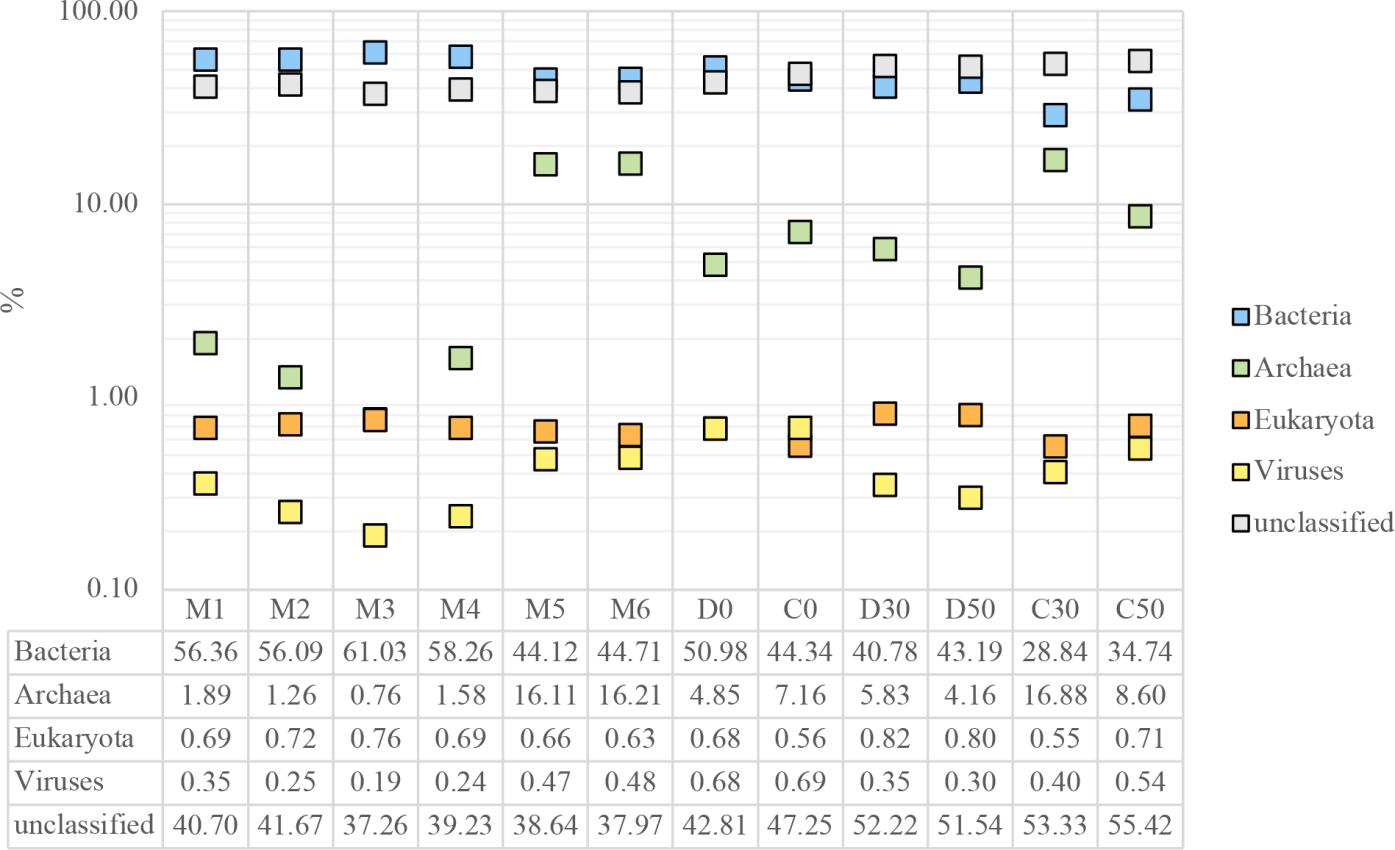
Percentage of AD reads assigned to Bacteria, Archaea, Viruses or unclassified. Surface samples from 2016 to 2018 (**m1-m4**) have a larger abundance of reads assigned to Bacteria and a lower abundance of reads assigned to Archaea and Viruses. Metagenomes sampled at depths of 30 and 50 cm (**d30, d50, c30, c50**) show the largest proportion of unclassified reads. Absolut values corresponding to 100 % are presented in Table 1.

To assess the factors these domain differences can be attributed, we compared the variations in relative abundance among samples grouped by season or depth. We could not find significant differences between dry (M1, M3 and M5) and wet (M2, M4 and M6) seasons samples. However, there were significant changes between some sample groups. For instance, surface samples from 2019 and 2020 (M5, M6, D0 and C0) are distinguished from surface samples from 2016 to 2018 (M1, M2, M3 and M4) by a lower proportion of reads assigned to Bacteria (t-Student, t=5.93, *P*=0.0041), coupled with a greater relative abundance of reads assigned to Archaea (*P*=0.0312) and Viruses (*P*=0.0098) (Fig. 2). Interestingly, surface samples from 2019 onwards only differ significantly from the depth samples (D30, D50, C30 and C50) by a lower percentage of unclassified reads (*P*=0.0077). However, until more samples are obtained for comparison, interpretations should be taken with reservation.

On average, the microbial community was dominated by bacteria of the phylum *Proteobacteria* (44.32 %), followed by *Bacteroidetes* (10.66%), *Cyanobacteria* (10.49%) and *Chloroflexi* (9.14%). *Cyanobacteria* reached their highest abundance in the shallow samples reaching dominance in M3 (45.08%), but suffering a large decrease in M5 and M6 (on average 1.96%) and in deep samples (on average 0.18 %). *Chloroflexi* maintained an abundance of less than 10 % in most samples but appeared to become more abundant with depth, reaching an average abundance of 34.26 % at D30 and D50. It is also important to note that *Euryarcaheota* had an abundance of less than 0.1 % in surface samples from 2016 to 2018 but reached an average abundance of 14.26 % at M5 and M6, after which their abundance never again became as low (Fig. S1).

The viral community was, on average, dominated by reads assigned to the order *Caudovirales*, namely to the families *Siphoviridae* (53.35 %), *Myoviridae* (31.48 %) and *Podoviridae* (11.92 %). These viral families are also ubiquitous in hypersaline sites [25] and other extreme environments, except for deep sea sediments and cold environments [51]. Viruses of the order *Caudovirales* – also known as head-tailed viruses – are the most abundant type of viruses on Earth [52], and are non-enveloped viruses with ichosahedral heads or capsids attached to a hollow flexible tail. They account for 96 % of viruses infecting bacteria [53] and the majority of viral isolates infecting halophilic or methanogenic euryarchaea, accounting for almost half of all archaeal viruses studied [54]. These proportions are somewhat similar to other CCB viromes [33], albeit with a lower proportion of viruses belonging to the family *Microviridae* (0.51%) and a subtle presence of *Herelleviridae* (1.15 %) and *Marseilleviridae* (1.56 %) (Fig. S2A).

At genus level, AD had a high abundance of *Donellivirus* (36.29 %), which includes *Bacillus phage G*, initially isolated from its host *Bacillus megaterium* [55], followed by *Ahduovirus* (17.74 %), which includes *Burkholderia phage vB_BceS_AH2*, originally isolated from plant-associated soil samples [56], *Barbavirus* (12 %), which includes various *Rheinheimera* sp. phages isolated from Baltic Sea samples [57], *Emdodecavirus* (11.12 %), including *Sinorhizobium phage phiM12* that infects *Sinorhizobium meliloti* [58], *Shapirovirus* (7.03 %), which includes various *Caulobacter crescentus* phages, some of which have been isolated from superficial freshwater samples [59], and *Bellamyvirus* (6.52 %), comprising the *Synechococcus phage Bellamy*. These are all head-tailed viruses which, as seen in Fig. S2B, can also be found in hypersaline viromes (*Donellivirus*, *Barbavirus* and *Bellamyvirus*), in other CCB sites (*Barbavirus*, *Embdodecavirus* and *Shapirovirus*) and ocean viromes (*Bellamyvirus*), except for *Ahduoviruses* which are rare or absent in other viromes. *Bacillus megatrium* and *Sinorhizobium meliloti* are present in the AD metagenomes as are numerous reads assigned to members of the genera *Burkholderia *, *Rheinheimera *, *Caulobacter* and *Synechococcus *, suggesting that the aforementioned viruses may indeed be present and infecting their corresponding hosts at this site.

**Fig. 3.**
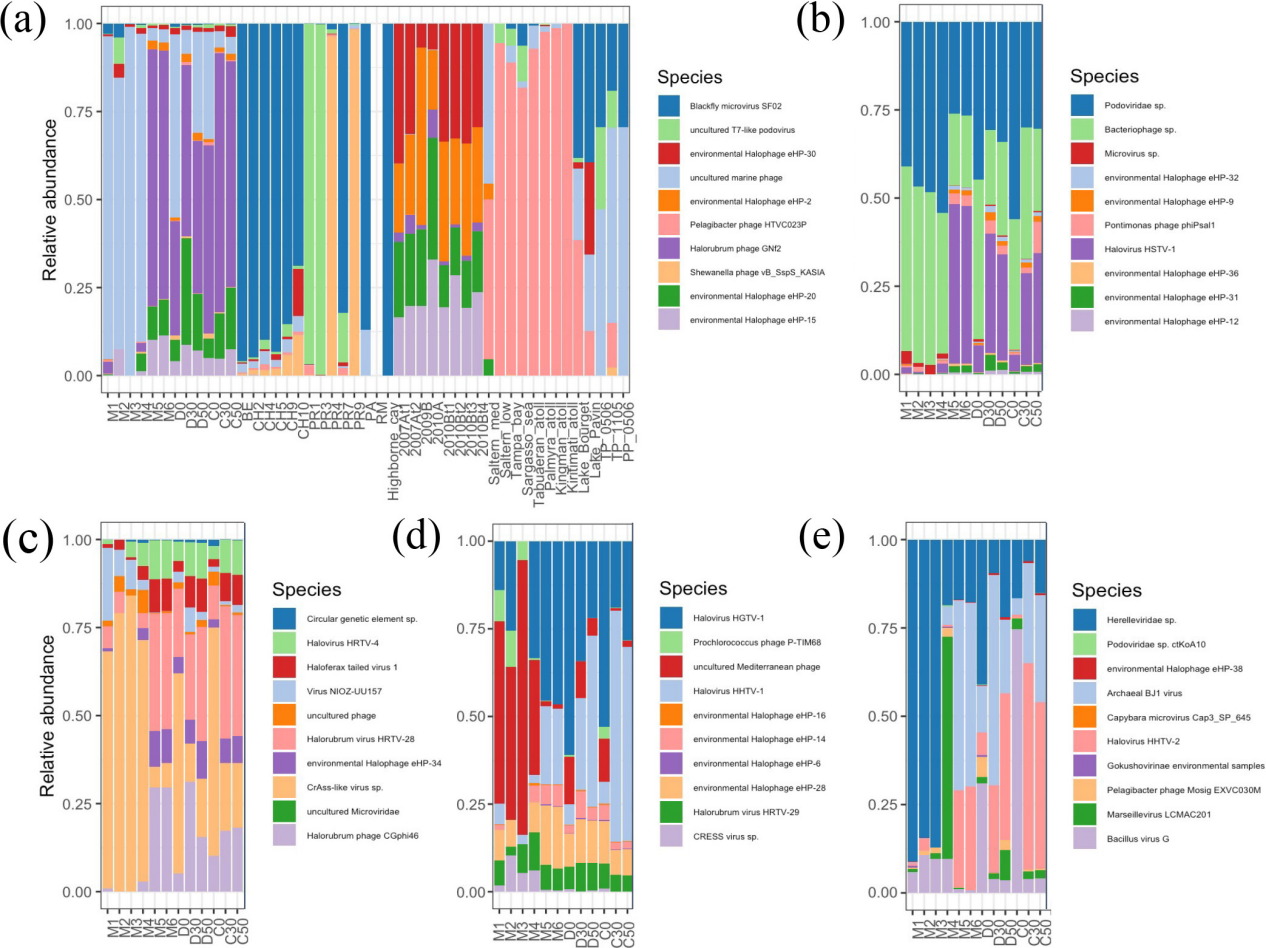
Relative abundance of reads assigned to viral species. For clarity, 91 species were sorted in descending order of mean relative abundance obtained from the 47 metagenomes, and are presented in separate graphs in groups of ten. (**a**) Fifth group of most abundant species for 12 AD metagenomes and 35 metagenomes from other environments (see Table S1). From M5 onwards, a high abundance of reads assigned to haloarchaeaviruses, which are also found in other hypersaline environments, can be observed. (**b**) Second group of most abundant species for 12 AD metagenomes. (**c**) Third group of most abundant species for 12 AD metagenomes. (**d**) Fourth group of most abundant species for 12 AD metagenomes. (**e**) Seventh group of most abundant species for 12 AD metagenomes. (b–e) show that M5 and M6 have abundances similar to those of 30 and 50 cm depth samples. Absolute values corresponding to 100 % range from 44 067 in 2009B to 97 972 in BE, with an average of 76 045. For AD metagenomes the variation is from 62 939 in D50 to 73 174 in M1, with an average of 69 740.

As seen in Fig. 2, late surface samples (M5, M6, D0 and C0) had a larger abundance of reads assigned to Archaea and Viruses when compared to early surface samples. This trend can be further explored for viral reads with successful taxonomy assignment at species level. For instance, from samples taken during 2019 (M5 and M6) onwards, an increase in reads assigned to haloarchaeaviruses also found at other hypersaline sites could be observed (Fig. 3a).

For surface samples from 2019 onwards, there was a considerable decrease in reads assigned to *Microviridae* sp., *Circoviridae* sp., *Microvirus* sp., Prokaryotic dsDNA virus sp., uncultured marine phage and *Synechococcus* phage S-SCSM1, which were also less abundant at the samples of 30 and 50 cm depth, coupled with a considerable increase in reads assigned to viruses that infect halophilic Archaea and some environmental halophages with no known host (eHP-6, eHP-15, eHP-20, eHP-28, eHP-31 and eHP-34) [53, 60], which are also more abundant in the 30 and 50 cm depth samples. In turn, 30 and 50 cm depth samples had a greater abundance of some reads assigned to viruses that infect halophilic Archaea compared to both surface samples from 2016 to 2018 and from 2019 onwards (Table S1). Although the number of samples in each sampling time group was low (*n*=3), the magnitude of the changes strongly suggests that the differences are significant.

**Fig. 4.**
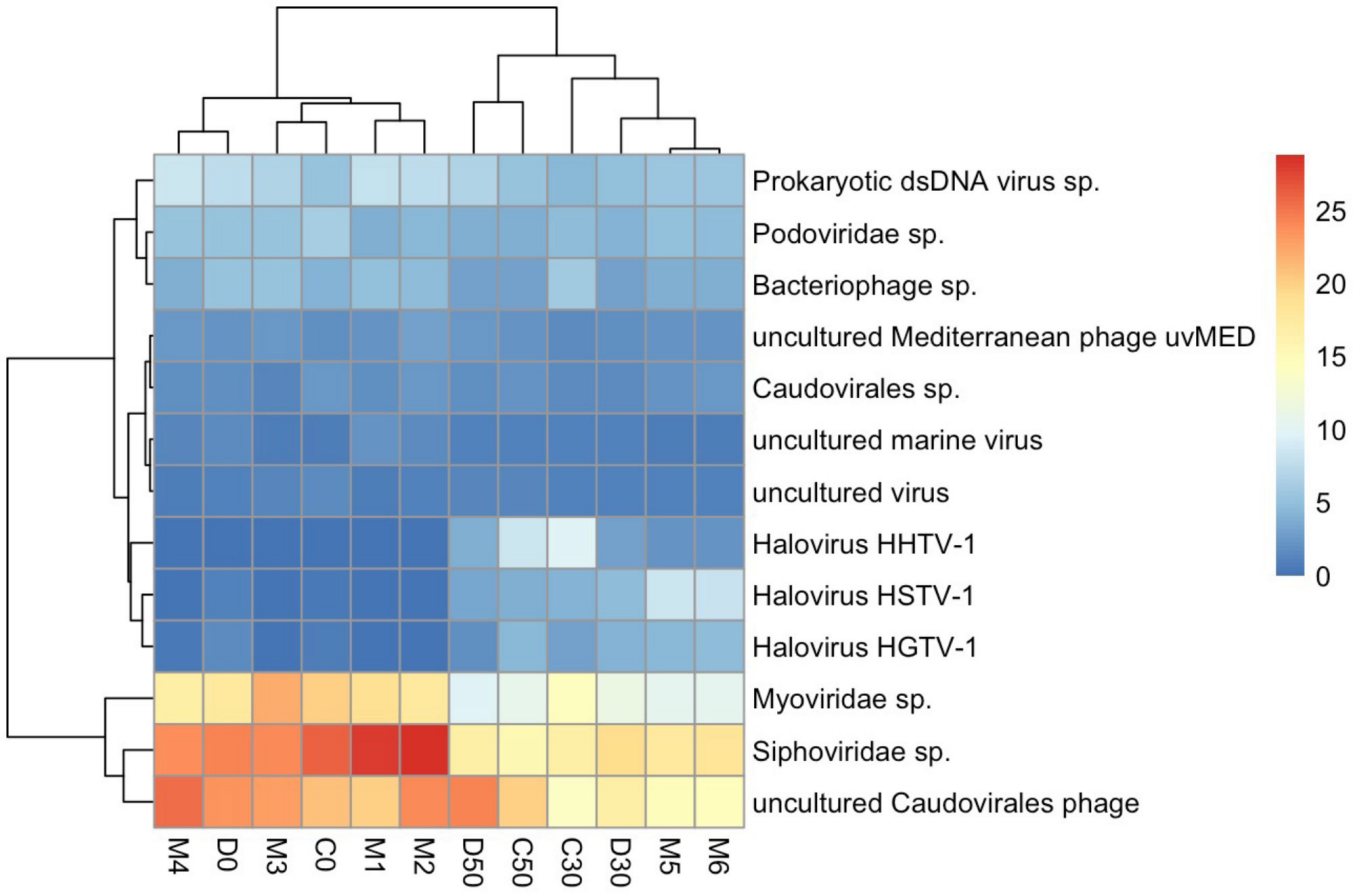
Hierarchical clustering based on relative abundance of reads assigned to viral species with at least 1 % average abundance across all AD metagenomes. Most surface samples cluster together, including D0 and C0 which are late surface samples (2020), except for M5 and M6 (2019) which cluster with 30 and 50 cm depth samples. Absolute values corresponding to 100 % range from 50 897 in D50 to 66 360 in M1, with an average of 60 028.

Interestingly, among surface samples from 2019 onwards, M5 and M6 showed haloarchaeavirus abundances more similar to those at 30 and 50 cm deep samples (Fig. 3b–e). To further explore this trend, we performed a hierarchical clustering based on the relative abundance of reads assigned to viral species with at least 1 % average abundance across all AD samples. This analysis suggested that the AD samples could be divided into two groups: the surface samples, including M1-M4, D0 and C0; and the deep samples (*sensu lato*), which included the 30 and 50 cm deep samples along with M5 and M6 (Fig. 4). A comparison between AD deep *sensu lato* and surface samples showed significant differences (t-Student, *P*<0.05) in 45 out of 86 OTUs with species assignment in AD viromes (Table S2). These are 12 OTUs more abundant in surface viromes, including cyanophages, and 33 OTUs more abundant in deep viromes *sensu lato*, including all OTUs assigned to haloviruses.

As seen in Table 2, surface samples from 2016 to 2018 are characterized by a low haloarchaeavirus abundance (< 2 %), while surface samples from 2019 to 2020 and deep samples have haloarchaeavirus abundance greater than 3 %. However, M5 and M6 reach the haloarchaeavirus abundance that can be found in the deep samples (> 20 %). On average, haloarchaeaviruses reach 15.37 % relative abundance, which is higher than what is found in low hypersaline sites (3%) but lower than what can be found in high hypersaline sites (up to 76 %) [25].

**Table 2.** AD samples with their sampling year, sampling depth, the number of reads assigned to viral species, number of reads assigned to haloarchaeavirus and the relative abundance of haloarchaeavirus. The relative abundance of haloarchaeaviruses ranges from 0.17 % in M3 to 32.20 % in C30

Sample	Year	Depth (cm)	Reads assigned to viral species	Reads assigned to haloarchaeaviruses	Haloarchaeavirus abundance (%)
M1	2016	Surface	73 174	652	0.89
M2	2016	Surface	68 923	321	0.47
M3	2017	Surface	70 741	123	0.17
M4	2018	Surface	67 637	1200	1.77
M5	2019	Surface	71 337	21 156	29.66
M6	2019	Surface	71 146	20 945	29.44
D0	2020	Surface	68 449	3752	5.48
D30	2020	30	69 594	21 088	30.30
D50	2020	50	62 939	12 913	20.52
C0	2020	Surface	71 094	2343	3.30
C30	2020	30	70 555	22 719	32.20
C50	2020	50	71 293	21 544	30.22

**Fig. 5.**
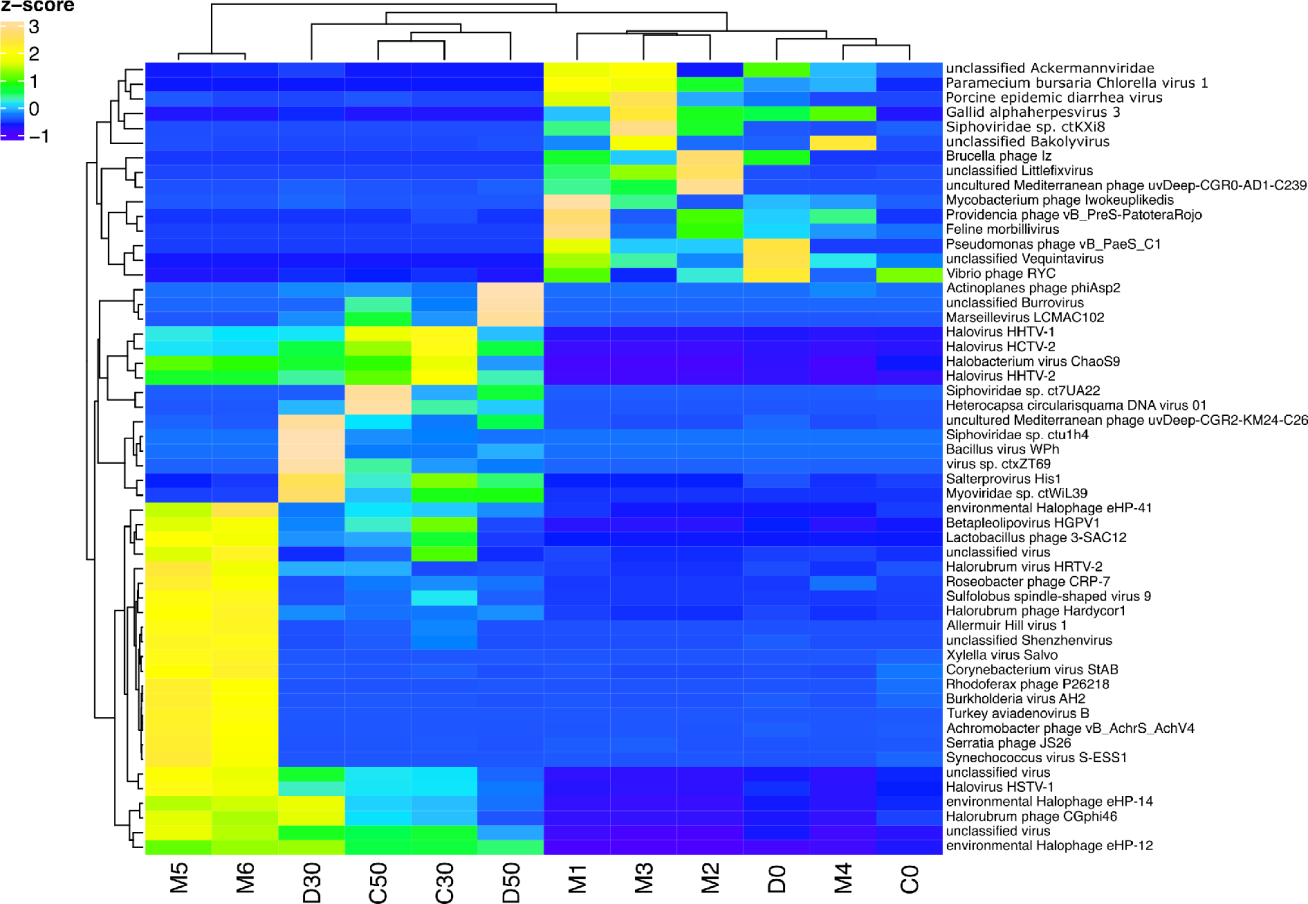
Heatmap representing differentially abundant viral OTUs of AD at CCB, using all OTUs to evaluate differential abundance between surface and deep samples (log fold change=1.5 and *p*-value=0.01) between deep (*sensu lato*) and surface samples.

Since these taxonomic analyses were based on the relative abundance of filtered abundant OTUs with successful taxonomic assignments (see Methods), we also performed a more exhaustive analysis, including all OTUs at AD to evaluate differential abundance between surface and deep *sensu lato* samples (an equivalent analysis was performed between dry and wet samples, showing no significant differentiation; analysis not shown). Briefly, we used the raw counts table and followed IDEAmex pipeline for differential expression analysis [49] with the package edgeR [50]. There were 54 out of 3228 differentially abundant OTUs (log fold change=1.5 and *p*-value=0.01). Of these, 39 were more abundant in deep samples, and 15 were more abundant in surface samples (Fig. 5). Of the abundant OTUs in deeper samples, 14 were assigned to archaeal viruses, of which 13 belonged to haloarchaeaviruses, and one to an acidophilic and hypertherophilic archaea virus. There were no OTUs assigned to haloviruses or viruses of archaea significantly more abundant in surface samples. Within OTUs with non-significant differential abundances, there were 98 haloviruses of which 95 showed a tendency to a greater abundance in deep samples. Interestingly, although M5 and M6 viromes were clearly differentiated from other surface viromes and shared some characteristics with deep viromes, there were some differentially abundant OTUs which were in low abundance in M5 and M6 (as in surface viromes) and others which were only abundant in M5 and M6 but not in depth or surface viromes.

Consistent with Table 2, 2020 surface viromes (D0 and C0) showed a less drastic decrease in OTUs assigned to haloviruses compared to other surface viromes. In fact, in a MDS of normalized counts M5 and M6 could be somewhat differentiated from both surface and depth viromes, and D0 and C0, although clustered with other surface viromes, were slightly closer to deep viromes (Fig. S3).

### Viral diversity in AD and comparison with other viromes

**Fig. 6.**
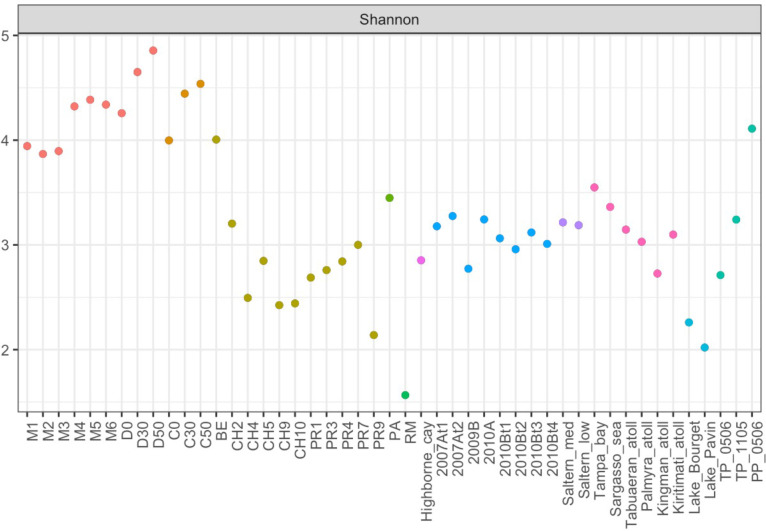
OTU level alpha diversity index (Shannon) for 12 viral metagenomes from AD, at CCB, and 35 metagenomes from other environments (see File S1). AD viromes are represented by red points. Other CCB viromes (PR and CH) are represented by olive green points. Sea viromes are represented by pink points. High hypersaline viromes are represented by blue points.

Within AD, Shannon’s diversity index was greater for 30 and 50 cm depth viromes in comparison to both early (t-Student, t=4.44, *P*=0.0044) and late (t-Student, t=3.04, *P*=0.0228) surface viromes (Fig. 6). Estimated Richness (Chao1) was significatively higher (t-Student, t=2.68, *P*=0.0368, Fig. S4A) for late 2019–2020 viromes of AD in comparison to early 2016–2018 AD viromes, while Evenness (Simpson) was greater (t-Student, t=9.9, *P*=6.12e-05, Fig. S4B) in 30 and 50 cm depth viromes compared to early 2016–2018 viromes.

Since, as shown above, the 2019 surface samples (M5 and M6) showed higher similarities with the 30 and 50 cm depth samples, we grouped the 2019 surface samples with the 30 and 50 cm depth samples (deep samples, *sensu lato*) and compared them against the rest of the surface samples. Both the Simpson and Shannon indices had higher values for deep viromes *sensu lato* in comparison to surface viromes (*P*=2.12e-07 and *P*=0.0014, respectively). There are no differences between dry and wet seasons.

Alpha diversity estimates showed that AD harbour the most diverse viral community among all other available viromes, including CCB, hypersaline, sea and freshwater samples (Shannon Fig. 6; Chao1 Fig. S4A and Simpson Fig. S4B, all at OTU level). More specifically, AD show significantly higher Shannon index values than those of Churince (t-Student, t=9.12, *P*=3.92e-05), Pozas Rojas (t-Student, t=9.33, *P*=3.38e-05), high hypersaline (t-Student, t=11.25, *P*=2.67e-09) and ocean (t-Student, t=7.75, *P*=8.79e-06) viromes.

**Fig. 7.**
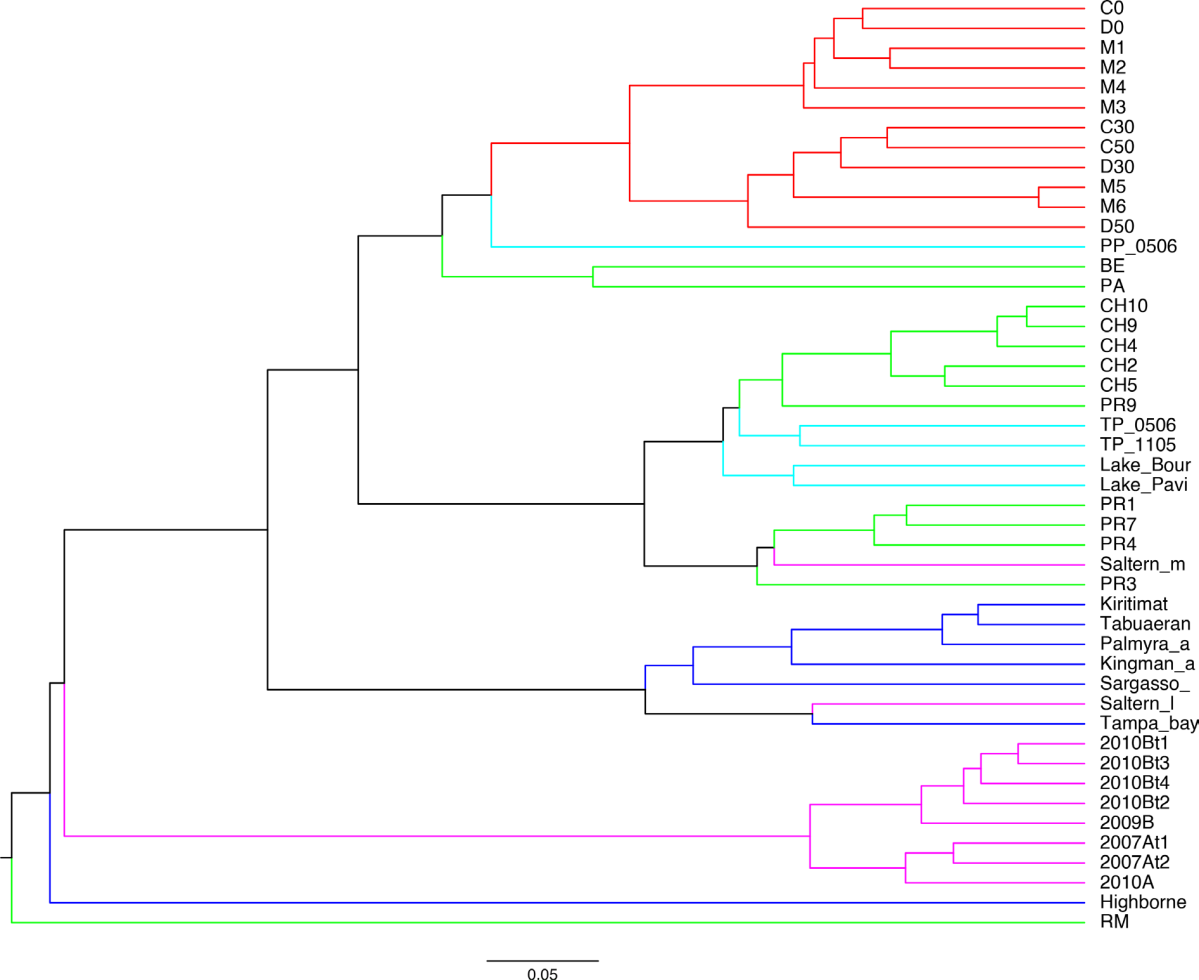
UPGMA tree computed from normalized species-level Bray-Curtis dissimilarity matrix. This matrix is based on the relative abundance of filtered abundant OTUs with successful taxonomic assignments. Branches leading to AD viromes are highlighted in red. Branches leading to other CCB viromes are highlighted in green. Freshwater, ocean and hypersaline viromes are highlighted by cyan, blue and magenta branches, respectively. This tree shows that AD harbours a unique viral community more similar to those from other CCB sites than to those from other hypersaline sites.

The Bray-Curtis clustering showed that AD viromes were more similar to other CCB viromes than to hypersaline viromes, and that AD formed a cluster of their own within which samples were grouped by depth rather than seasons (Fig. 7). Consistent with previous groupings, M5 and M6 were clustered with 30 and 50 cm deep viromes, while 2020 surface viromes (D0 and C0) were clustered with the early AD surface viromes.

A NMDS analysis based on Bray-Curtis dissimilarities (Fig. S5) between the 47 viromes, supported the analysis described above, showing that all AD viromes shared a unique viral community despite seasonal differences in pH and salinity and that neither AD viromes derived from saturation conditions during the dry season cluster with other highly hypersaline viromes, nor do AD viromes derived from lower (5 %) hypersaline conditions during the wet season clustered with other low or intermediate hypersaline viromes.

**Fig. 8.**
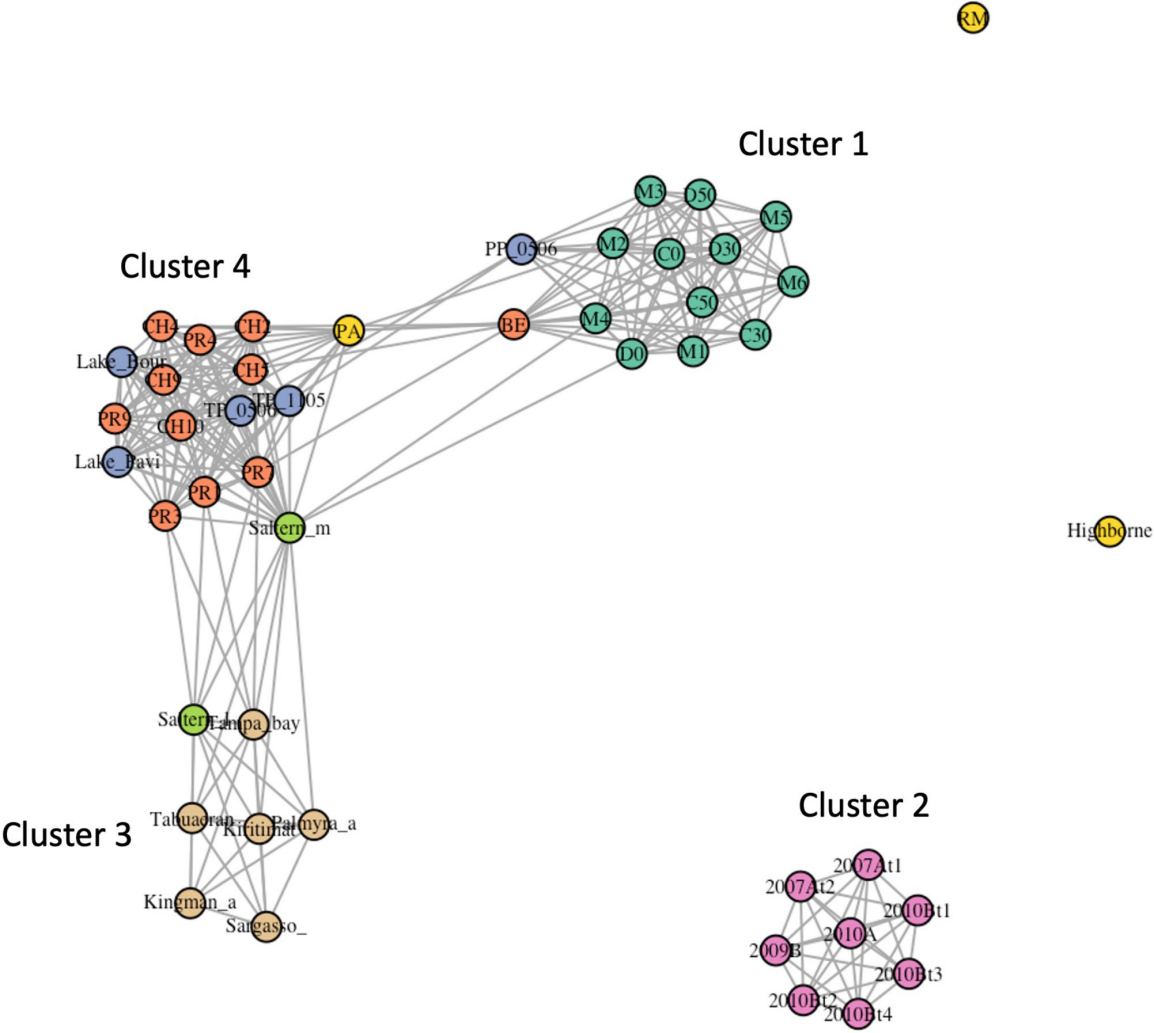
OTU level Bray-Curtis similarity network showing 25 % (above third quartile) of the strongest similarities. AD viromes are represented by jade green circles within Cluster 1. Other CCB viromes (PR and CH) are represented by orange circles within Cluster 4. Ocean viromes are represented by beige circles within Cluster 3. High hypersaline viromes are represented by pink circles within Cluster 2.

For a more in-depth analysis, we transformed the OTU level Bray-Curtis dissimilarity matrix into a similarity matrix (1 – dissimilarity) and visualized the similarity distribution on networks built with the igraph library [61], as shown in Fig. 8. Within the 15.9 % (above one standard deviation) of the strongest similarities, four different clusters could be found: Cluster 1, including all AD viromes; Cluster 2, including all high hypersaline viromes; Cluster 3, with all ocean viromes plus the low salinity hypersaline virome and; Cluster 4, with the other CCB viromes (all from Churince and Pozas Rojas) along with four freshwater viromes. In Cluster 4 Pozas Rojas viromes are also connected to the intermediate salinity hypersaline virome and one Churince virome is also connected to Pozas Azules and La Becerra viromes. Within the 25 % (above third quartile) of the strongest similarities, high-salinity hypersaline viromes remained as an isolated cluster, while AD viromes showed connections with Cluster 4, namely between AD surface and Churince viromes (Fig. 8). At this point, Cluster 3 was also connected to Cluster 4 through Pozas Rojas and intermediate salinity hypersaline viromes.

When the top 50 % (above second quartile) similarities were considered (Figure not shown), Cluster 1 and Cluster 4 appeared as a tightly packed cluster with a weaker association to Cluster 3 (via Pozas Rojas viromes) and no association to Cluster 2. If the top 75 % (above first quartile) similarities were considered, Cluster 1, Cluster 4 and Cluster 3 merged as a single cluster with a weaker similarity to Cluster 2 mainly due to AD deep viromes (Fig. S6).

Overall, CCB viromes (Churince and Pozas Rojas) were similar to freshwater viromes, especially those from Churince (average Bray-Curtis=0.3423). Also, AD viromes were more closely related to Churince (average Bray-Curtis=0.6378), Pozas Rojas (average Bray-Curtis=0.6607) and freshwater viromes (average Bray-Curtis=0.6863), than to oceanic (average Bray-Curtis=0.7440), and high salinity hypersaline viromes (average Bray-Curtis=0.8788). Interestingly, Pozas Rojas viromes were more closely related to oceanic viromes (average Bray-Curtis=0.6285) than to those from AD. Other studies in CCB have described a high β-diversity for viral [32, 33] and microbial [62] communities. However, those studies either lacked appropriate methods for comparison or did not include enough metagenomes from other environments to establish a scale that would put diversity differences between different CCB sites into context. More recent studies have recognized that CCB viral communities had similarities to some marine communities, as previously described [32], and some freshwater communities [33], and a close relationship between Churince and Pozas Rojas microbial communities, with some Pozas Rojas samples closely related to the epipelagic zone of the Mediterranean Sea [63]. Here we showed that, although highly diverse, CCB environments may actually harbour a group of somewhat related microbial communities.

Another interesting trend is that AD viromes are the least dissimilar from high salinity hypersaline viromes. More strikingly, viromes from deeper samples (including M5 and M6) were significatively less dissimilar (*P*=3.6e-32) from high salinity hypersaline viromes (average Bray-Curtis=0.8354, *n*=48) than surface viromes (average Bray-Curtis=0.9223, *n*=48). Deep viromes *sensu stricto* were also significatively less dissimilar (*P*=6.26e-05) from high salinity hypersaline viromes (average Bray-Curtis=0.8348, *n*=32) than late surface viromes (average Bray-Curtis=0.8692, *n*=32) which in turn were less dissimilar (*P*=4.17e-11) than early surface viromes (average Bray-Curtis=0.9326, *n*=32). This trend highlights the fact that, although not closely related to viral communities from other high hypersaline sites, AD is a hypersaline site harbouring a considerable abundance of halarchaeaviruses that become more abundant and diverse at greater depths.

Given that AD harbours microbial mats associated with elevated salinity and pH, it is likely that the viral community resembles that of other hypersaline microbial mats or that of soda lakes. To test this hypothesis, we downloaded five metagenomes from hypersaline microbial mats and soda lakes, respectively (Table S3), and ran the COMETS pipeline (see Methods) to conduct an alpha and beta diversity analysis in perspective with all previous metagenomes. The OTU level Bray-Curtis similarity network showing 25 % (above third quartile) of the strongest similarities (Fig. S7) had an overall structure very similar to that of Fig. 8, except that high hypersaline viromes were now connected to the rest of the viromes through soda lakes viromes. Two soda lake viromes appear closely related to viromes from AD (HC26S and Wadi El-Natrun). However it is four hypersaline microbial mat viromes from other sites (Great Salt Lake, Tristomo elos1, Tristomo elos7 and Tristomo elos12) that appear to group within the AD cluster. Interestingly, these two viromes from soda lakes and four from hypersaline microbial mats, showed alpha diversity indices as high as those from AD (Fig. S8).

Interestingly, the high diversity of soda lakes has inspired the soda ocean hypothesis [24], according to which conditions on the early Earth would have allowed the formation of an alkaline ocean that would have favoured some of the reactions essential for the formation of life and the proliferation of stromatolite-forming organisms [19, 64].

### Where does AD viral diversity come from?

The Grinnellian niche concept states that community structure is driven by environmental variables [65, 66]. Hypersaline viral communities have been shown to follow global patterns such that their structure and diversity are driven by changes in salinity levels [25]. Therefore, given the high salinity and the fluctuating conditions of the analysed samples from the Archaean Domes (AD) in the Cuatro Cienegas Basin (CCB), we expected to find a viral community whose structure and diversity, and its response to changes in salinity, would resemble those from other hypersaline sites. We found a community dominated by viruses belonging to the order *Caudovirales* (Fig. S2A), as would be expected from other hypersaline environments [25] and also from other environments from temperate to extreme [51], and a considerable abundance of reads assigned to haloarchaeaviruses (Table 2). Neither the community structure (Fig. 3) nor the alpha diversity (Fig. 6) in AD viral community are driven by environmental fluctuations. For instance, neither samples from the wet season group with low or intermediate salinity hypersaline viromes, nor samples from dry season group with other high hypersaline viromes (Fig. S5). Instead, AD viromes form a cluster of their own, within which the subgroups are sorted by depth rather than season (Fig. 4, Fig. 7). Also, in contradiction to what has been reported in viral communities from other hypersaline sites (regarding the decrease in diversity when the abundance of haloarchaeaviruses is high) [25], in AD samples – in which there is a higher abundance of haloarchaeavirus – higher viral diversity was also observed (Fig. 6). A similar trend with increased microbial diversity at higher salinity, pH and depth has been reported in Ethiopian soda lakes [67].

If environmental variables are not the main community drivers, it is possible that the community dynamics fit an Eltonian niche concept, which states that community structure is driven by interactions [63, 65]. For instance, considering that naked haloarchaeaviruses are only indirectly affected by changes in salinity [53], that AD viral community is dominated by viruses belonging to the order *Caudovirales*, which are naked viruses, and that a highly diverse and seasonally stable core microbial community has been recently described in AD [14], one could argue that AD viral community will tend to remain stable as long as the host community remains the same. This may be related to the so called ‘insurance hypothesis’, which predicts that highly diverse ecosystems remain functionally stable in changing environments [66]. In addition, organisms inhabiting AD should probably be poikilotrophic, i.e. poly-extremophiles adapted to an environment subject to extreme and sporadic physicochemical changes [68]. Such may be the case for soda lakes inhabited by microorganisms adapted to both elevated pH and salinity. For example, the highly diverse microbial communities of the Kulunda steppe soda lakes, where it has been argued that environmental fluctuations (salinity) promote the maintenance of high diversity [23].

One possible evidence of virus-host interactions in AD is the relatively high abundance of reads assigned to viruses infecting Archaea, which is consistent with the high abundance and diversity of Archaea reported in previous studies [9, 10]. Also, the increase in Archaea abundance to 16 % since 2019 may be associated with an increase in virus abundance (Fig. 2), which in turn may contribute to the high diversity and stability of the microbial community via ‘kill the winner’ interactions [27, 30]. However, further analyses are needed to test the extent and relevance of virus-host interactions in this site.

Most CCB environments have a low carbonate alkalinity [69] and high Mg^2+^ and Ca^2+^ [69–71] which, despite being athalassic, is very similar to seawater ionic composition [70] where Mg^2+^ and Ca^2+^ concentrations are much higher than that of carbonates [20]. Given that AD viral community is closely related to that of other CCB environments (Fig. 7) we could expect the ionic composition to be similar to that of seawater. However, carbonate and bicarbonate measurements on 2019 AD samples resulted in a higher total alkalinity (TA=2[CO_3_^2-^] + [HCO_3_^-^]) (from 11.08 mmol l^−1^ during wet season to 32.75 mmol l^−1^ during dry season) in comparison to seawater (2.33 mmol l^−1^) [20] and other CCB sites (from ~0.5 mmol l^−1^ to ~6 mmol l^−1^) [70], but not as high as in Lake Van (~150 mmol l^−1^), which is the world’s largest soda lake [20]. Such alkalinity may be enough to speculate that AD is a soda lake, however this possibility cannot be confirmed until a full anionic/cationic analysis including Mg^2+^ and Ca^2+^ concentrations is made, in order to test the soda lake criterion (TA>2[Mg^2+^] + 2[Ca^2+^]) [20].

High salinity and pH (up to 9.5 during the wet season), as well as similar community diversity and composition to that of a high hypersaline sediment sample from Hutong Qagan soda lake (Inner Mongolia) (Fig. S7), add to the possibility that AD is a soda lake. However, soda lakes are considered the most stable high pH environments on Earth [19, 24], due to the buffering effect against strong pH variations conferred by high alkalinity [72], which contrasts with the drop in pH to 5.5 reported in AD during the dry season [9], when pH would be expected to increase if it were a true soda lake [20]. In these alkalinity-limited environments, the elevated pH may be the result of net CO_2_ removal by photosynthesis during the day. During the night, when there is no photosynthesis, CO_2_ is returned to the water through respiration and pH decreases again [72, 73]. Since all samples were taken during the day, we cannot know if this is the case for AD, however it could be a strong possibility since the highest pH is observed during the wet season when photosynthetic cyanobacteria proliferate. Another process that could explain the relatively higher alkalinity and pH at AD compared to other CCB sites is sulphate reduction, which is a proton-consuming process carried out by sulphate-reducing bacteria. Briefly, as sulphate reduction occurs, cyanobacterial mats are degraded and organic matter oxidizes, which results in chlorophyll Mg^2+^ solubilization and bicarbonate production, respectively [73–75]. Both, photosynthetic and sulphate-reducing metabolisms have been detected in AD [14].

The results presented here are in better agreement with the alternative hypothesis that the AD viral community will be more similar to that of other CCB sites due to their shared geological history and deep aquifer. Briefly, after the Pangea breakup, all northern Mexico was covered by a shallow sea that began to regress in the late Cretaceous due to the Laramide Orogeny, completing its regression and the isolation of the CCB from the Gulf of Mexico with the uplift of the Sierra Madre Oriental in the early Eocene [2, 3, 6]. In addition, isotopic studies have shown that deep aquifer groundwater is an important source for CCB aquatic systems [8], suggesting that the deep aquifer has preserved the conditions of an ancient ocean and has maintained ancient microbial lineages isolated from their marine relatives for millions of years, which has been long enough to allow for such a great microbial diversity to emerge [7, 8].

Although CCB microbial and viral communities have been shown to have high α- and β-diversity indices [33, 62], the clustering analyses of the viral communities presented here (Fig. 7) suggest that CCB microbial communities represent a set of related communities. In addition, the fact that AD viral community forms a cluster of its own, within which the subgroups are sorted by depth rather than season (Figs 4 and 7), that diversity increases at greater depths (Fig. 6), and that late surface samples (2019–2020) present traits akin to deep samples (Figs 3 and 5), suggest that the deep aquifer beneath AD harbours a highly diverse microbial community that is sporadically transported to the surface during water upwelling events (maybe moved by the magmatic pouch in the depths of Sierra San Marcos y Pinos [8]). Finally, given that deep viromes show the highest percentage of unclassified reads (Fig. 2), it is likely that the microbial community in the deep aquifer is largely constituted by still unknown microorganisms.

## Conclusions

Overall, these results show that AD harbour a highly diverse and unique viral community rich in haloarchaeaviruses. Although the presence of haloarchaeaviruses is unique for known CCB viromes, the community is still more similar to CCB viral communities than to those of other hypersaline sites, except for other hypersaline microbial mats.

AD are also distinguished from other hypersaline sites by the maintenance of high diversity despite increases in salinity and abundance of haloarchaeaviruses. In fact, AD diversity seems to be higher than in other environments around the world, except for other hypersaline microbial mats and some soda lakes, regardless of the season.

The uniqueness of this viral community is likely related to the great Archaea diversity and virus-host interactions that need further exploration to fully characterize the community dynamics of this exceptional site.

Finally, the similarities between late 2019–2020 surface viromes with depth viromes, which are highly diverse and rich in haloarchaeavirues, supports a hypothesis where hydrological processes such as upwelling of the deep aquifer can function as a ‘seed bank’ with great microbial diversity.

## Supplementary Data

Supplementary material 1

Supplementary material 2
