# Supplementary material for: Metagenomic comparisons reveal a highly diverse and unique viral community in a seasonally fluctuating hypersaline microbial mat
Source: Microb Genom. 2023 Jul 17;9(7):mgen001063. doi: 10.1099/mgen.0.001063 (PMC10438804; doi:10.1099/mgen.0.001063)
Supplement: Supplementary material 1 [file mgen-9-1063-s001.pdf]

SPECIES-LEVEL TAXONOMIC ASSIGNMENT	EARLY-LATE	EARLY-DEPTH	LATE-DEPTH
MICROVIRIDAE SP.	0.0015	0.0004	0.2566
CIRCOVIRIDAE SP.	0.0081	0.0106	0.2842
MICROVIRUS SP.	0.0153	0.0157	0.3708
PROKARYOTIC DSDNA VIRUS SP.	0.0231	0.0064	0.1933
UNCULTURED MARINE PHAGE	0.0394	0.0339	0.4209
HALOVIRUS HGTV-1	0.0199	0.0017	0.3799
HALORUBRUM PHAGE GNF2	0.0224	0.0004	0.3006
HALORUBRUM VIRUS HRTV-28	0.0327	0.0001	0.1236
ENVIRONMENTAL HALOPHAGE EHP-32	0.0292	0.0013	0.0932
ENVIRONMENTAL HALOPHAGE EHP-2	0.0353	0.0005	0.2949
ENVIRONMENTAL HALOPHAGE EHP-15	0.0372	0.0008	0.4660
ENVIRONMENTAL HALOPHAGE EHP-31	0.0375	0.0065	0.1457
ARCHAEAL BJ1 VIRUS	0.0390	0.0106	0.2793
ENVIRONMENTAL HALOPHAGE EHP-34	0.0396	0.0002	0.2539
ENVIRONMENTAL HALOPHAGE EHP-9	0.0413	0.0014	0.1511
ENVIRONMENTAL HALOPHAGE EHP-28	0.0443	0.0001	0.3291
HALORUBRUM PHAGE CGPHI46	0.0445	0.0212	0.3167
ENVIRONMENTAL HALOPHAGE EHP-6	0.0448	0.0001	0.1952
HALOVIRUS HRTV-4	0.0475	0.0015	0.1773
ENVIRONMENTAL HALOPHAGE EHP-14	0.0496	0.0194	0.2808
SYNECHOCOCCUS PHAGE S-SCSM1	0.0039	0.0363	0.0445
ENVIRONMENTAL HALOPHAGE EHP-11	0.0283	0.0035	0.0427
ENVIRONMENTAL HALOPHAGE EHP-20	0.0329	0.0061	0.0301
HALORUBRUM VIRUS HRTV-29	0.0375	0.0001	0.0443
HALOVIRUS HCTV-2	0.0411	0.0004	0.0017
HALOVIRUS HHTV-2	0.0534	0.0011	0.0145
ENVIRONMENTAL HALOPHAGE EHP-12	0.0569	0.0002	0.0297
HALOVIRUS HHTV-1	0.0570	0.0065	0.0163
ENVIRONMENTAL HALOPHAGE EHP-16	0.0718	0.0116	0.0241
ENVIRONMENTAL HALOPHAGE EHP-24	0.0908	0.0025	0.0030
PODOVIRIDAE SP. CTPVR23	0.1054	0.0127	0.0177
ENVIRONMENTAL HALOPHAGE EHP-38	0.2002	0.0001	0.0002

Supplemental table 1 Species-level t-Student p-values between early (n = 3), late (n = 3) and depth samples (n = 3). Only significant p-values are colored. Blue and red text designate a significatively greater or lower relative abundance for the reference sample, respectively.

SPECIES-LEVEL TAXONOMIC ASSIGNMENT	SURFACE	DEPTHS	P
SIPHOVIRIDAE SP.	25.8329	17.4080	2.0055E-05
UNCULTURED CAUDOVIRALES PHAGE	22.7780	17.4134	0.00984391
MYOVIRIDAE SP.	18.8575	11.2481	3.7883E-05
PROKARYOTIC DSDNA VIRUS SP.	7.2790	5.4325	0.00535573
BACTERIOPHAGE SP.	4.7722	3.7516	0.04271066
HALOVIRUS HSTV-1	0.3212	5.5070	0.00026996
HALOVIRUS HHTV-1	0.0552	4.9855	0.00377766
HALOVIRUS HGTV-1	0.5087	3.7021	0.00016193
UNCULTURED MARINE VIRUS	1.3306	0.9097	0.04508303
CRASS-LIKE VIRUS SP.	1.2643	0.6321	0.00290447
HALORUBRUM PHAGE GNF2	0.1797	1.7171	2.8321E-05
HALORUBRUM VIRUS HRTV-28	0.1510	1.6146	7.647E-07
UNCULTURED MARINE PHAGE	1.5131	0.2267	0.0249969
HALOVIRUS HCTV-2	0.0500	1.7151	0.00118913
ENVIRONMENTAL HALOPHAGE EHP-28	0.1048	1.3580	9.9806E-06
HALORUBRUM PHAGE CGPHI46	0.0654	1.2633	0.00123251
ARCHAEAL BJ1 VIRUS	0.0880	1.1582	0.00044929
HALOVIRUS HHTV-2	0.0340	1.0875	0.00017474
HALORUBRUM VIRUS HRTV-29	0.0836	0.7230	1.6945E-06
PONTIMONAS PHAGE PHIPSAL1	0.0669	0.5484	0.00308692
HALOVIRUS HRTV-4	0.0697	0.5177	2.1183E-05
ENVIRONMENTAL HALOPHAGE EHP-14	0.0320	0.4955	0.00103097
HALOFERAX TAILED VIRUS 1	0.0396	0.4519	1.8172E-05
ENVIRONMENTAL HALOPHAGE EHP-20	0.0405	0.4418	0.0017202
ENVIRONMENTAL HALOPHAGE EHP-34	0.0378	0.4302	5.4202E-06
ENVIRONMENTAL HALOPHAGE EHP-31	0.0239	0.3461	0.00024614
MICROVIRIDAE SP.	0.2977	0.0489	0.00123602
ENVIRONMENTAL HALOPHAGE EHP-15	0.0263	0.2294	0.00013617
ENVIRONMENTAL HALOPHAGE EHP-9	0.0222	0.2270	2.1266E-05
ENVIRONMENTAL HALOPHAGE EHP-11	0.0196	0.2131	0.00034844
MICROVIRUS SP.	0.1641	0.0299	0.01894391
ENVIRONMENTAL HALOPHAGE EHP-32	0.0204	0.1645	3.5596E-05
ENVIRONMENTAL HALOPHAGE EHP-12	0.0060	0.1192	3.4201E-06
ENVIRONMENTAL HALOPHAGE EHP-36	0.0050	0.0793	0.00038805
ENVIRONMENTAL HALOPHAGE EHP-2	0.0043	0.0570	1.8936E-05
ENVIRONMENTAL HALOPHAGE EHP-30	0.0197	0.0411	0.01863032
PROCHLOROCOCCUS PHAGE P-TIM68	0.0344	0.0048	0.00637372
ENVIRONMENTAL HALOPHAGE EHP-6	0.0010	0.0251	1.1884E-06
SYNECHOCOCCUS VIRUS BELLAMY	0.0189	0.0057	0.0377621
ENVIRONMENTAL HALOPHAGE EHP-16	0.0027	0.0205	0.00726656
BORDETELLA VIRUS PHB04	0.0035	0.0174	0.0462241
ENVIRONMENTAL HALOPHAGE EHP-24	0.0005	0.0194	0.01393725
PODOVIRIDAE SP. CTPVR23	0.0019	0.0182	0.03736158
ENVIRONMENTAL HALOPHAGE EHP-38	0.0009	0.0121	0.00169676
SPHAEROTILUS PHAGE VB_SNAP-R1	0.0005	0.0039	0.04272228

Supplemental table 2 Species-level average relative abundance and t-Student p-values of OTUs significatively more abundant (blue text) or less abundant (red text) in surface samples (n = 6) compared to deep samples *sensu lato* (n = 6).

**Sample**	**Run**	**Location**	**Sample Type**	**Reads**	**Reference**
Great_salt_lake	SRR10846467	Bridger bay, Great salt lake, Utah	Hypersaline microbial mat	19,922,338	Kanik et al. 2020
Hot_lake	SRR5271190	Hot Lake, Washington, USA	Hypersaline microbial mat	30,318,005	Lindemann et al. 2013
Tristomo_elos12	ERR6290777	Tristomo bay (Karpathos, Greece)	Hypersaline microbial mat	111,720,365	Pavloudi et al. 2022
Tristomo_elos1	ERR6290772	Tristomo bay (Karpathos, Greece)	Hypersaline microbial mat	134,529,087	Pavloudi et al. 2022
Tristomo_elos7	ERR6290775	Tristomo bay (Karpathos, Greece)	Hypersaline microbial mat	144,236,134	Pavloudi et al. 2022
DK32S	SRR9330145	Habor Lake (Inner Mongolia Autonomous Region, China)	Soda lake	67,353,198	Zhao et al. 2020
HC22W	SRR9330148	Hutong Qagan Lake (Inner Mongolia Autonomous Region, China)	Soda lake	47,829,334	Zhao et al. 2020
HC26S	SRR9330142	Hutong Qagan Lake (Inner Mongolia Autonomous Region, China)	Soda lake	56,302,065	Zhao et al. 2020
HC5W	SRR9330150	Hutong Qagan Lake (Inner Mongolia Autonomous Region, China)	Soda lake	52,132,576	Zhao et al. 2020
Wadi_El-Natrun	ERR1770058	Wadi El-Natrun, Egypt	Soda lake	19,989,051	ZeinEldin et al. 2023

Supplemental table 3 Samples used to compare AD viral community with that of hypersaline microbial mats and soda lakes.

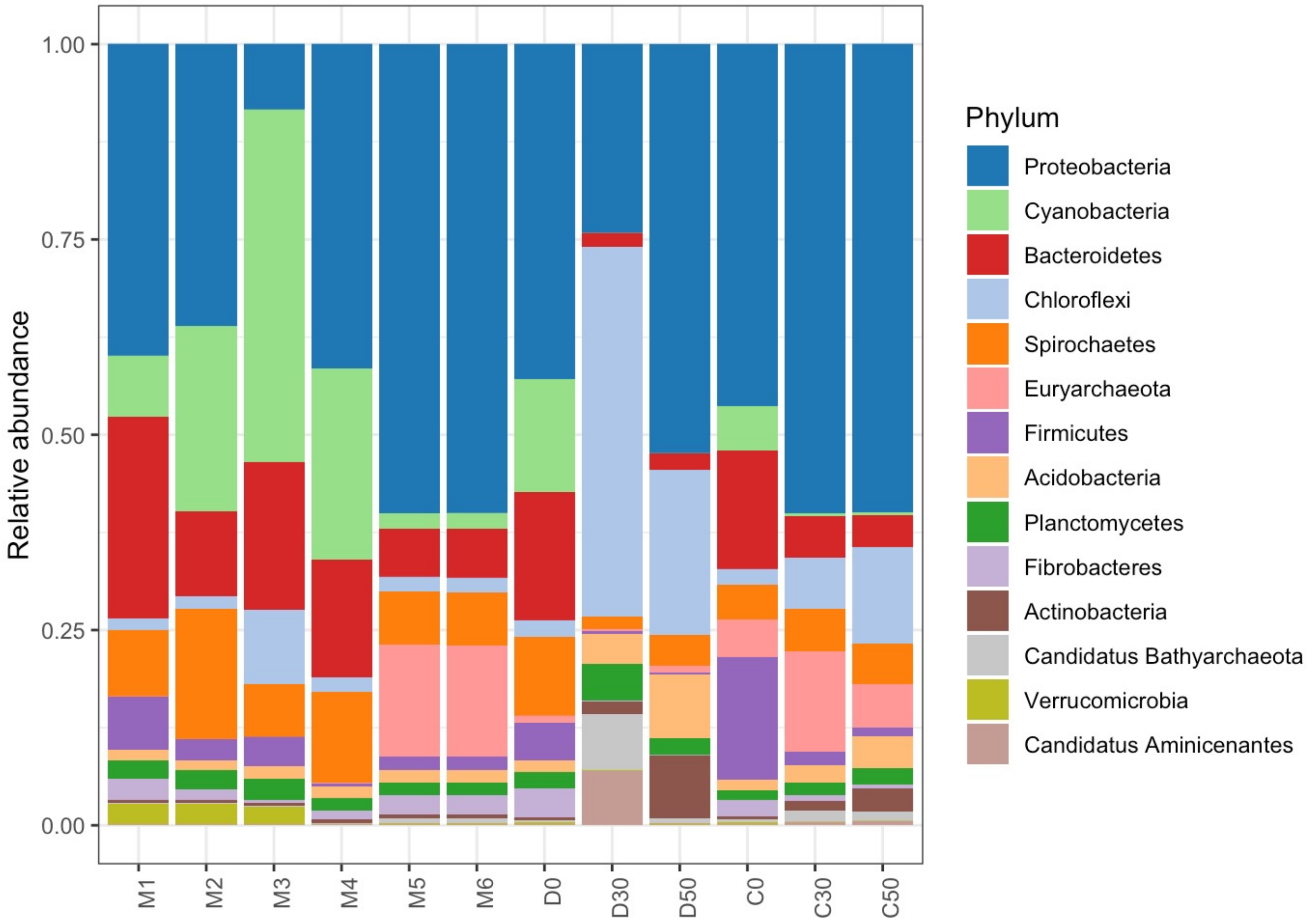

Supplementary figure 1 Relative abundance of reads assigned to bacterial and archaeal phyla for 12 AD metagenomes.

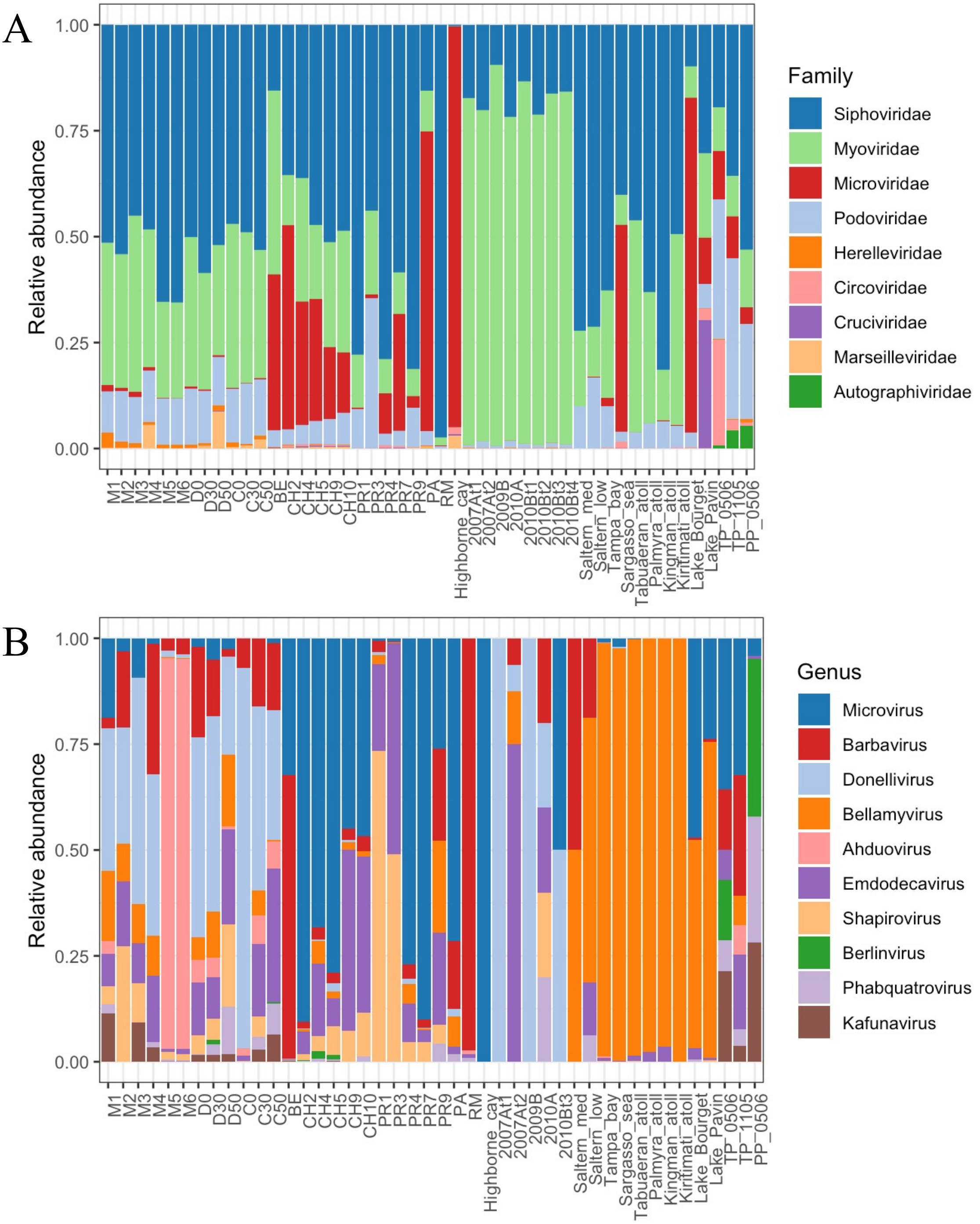

Supplementary figure 2 Relative abundance of reads assigned to viral families (A) and genera (B) for 12 AD metagenomes and 35 metagenomes from other environments (see Supplementary table 1).

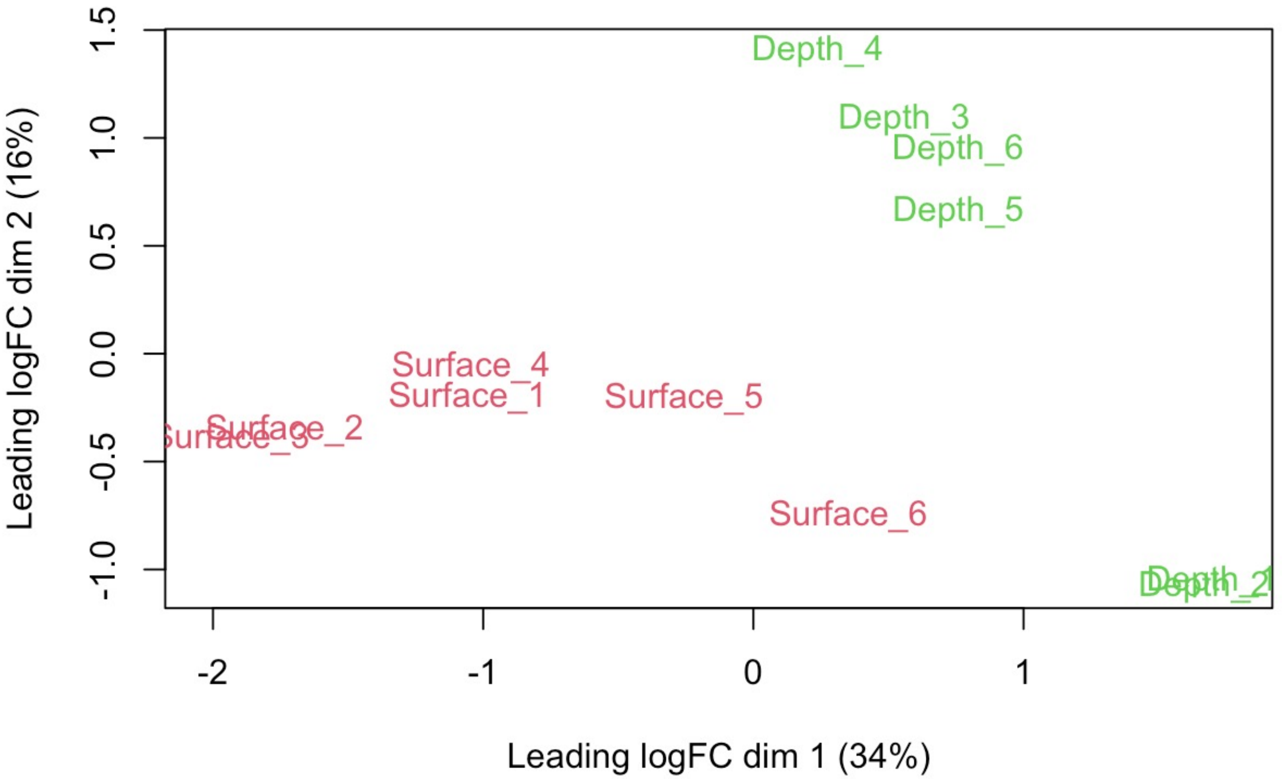

Supplementary figure 3 MDS analysis of all OTUs normalized counts. Surface_1 = M1, Surface_2 = M2, Surface_3 = M3, Surface_4 = M4, Surface_5 = D0, Surface_6 = C0, Depth_1 = M5, Depth_2 = M6, Depth_3 = D30, Depth_4 = D50, Depth_5 = C30, Depth_6 = C50.

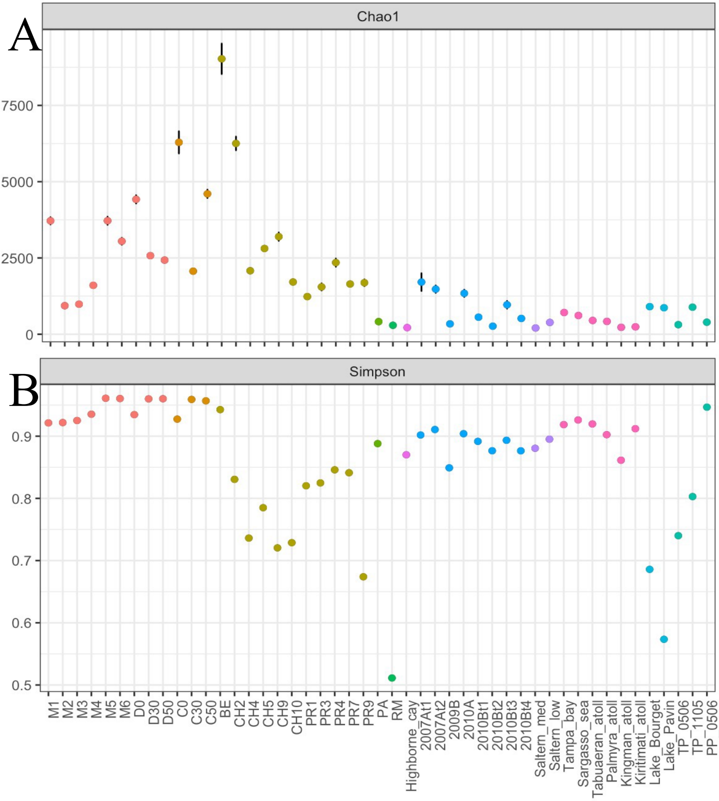

Supplemental figure 4 Chao1 (A) and Simpson (B) diversity indexes for 12 AD metagenomes and 35 metagenomes from other environments (see Supplemental File 1). AD viromes are represented by red points. Other CCB viromes (PR and CH) are represented by olive green points. Sea viromes are represented by pink points. High hypersaline viromes are represented by blue points.

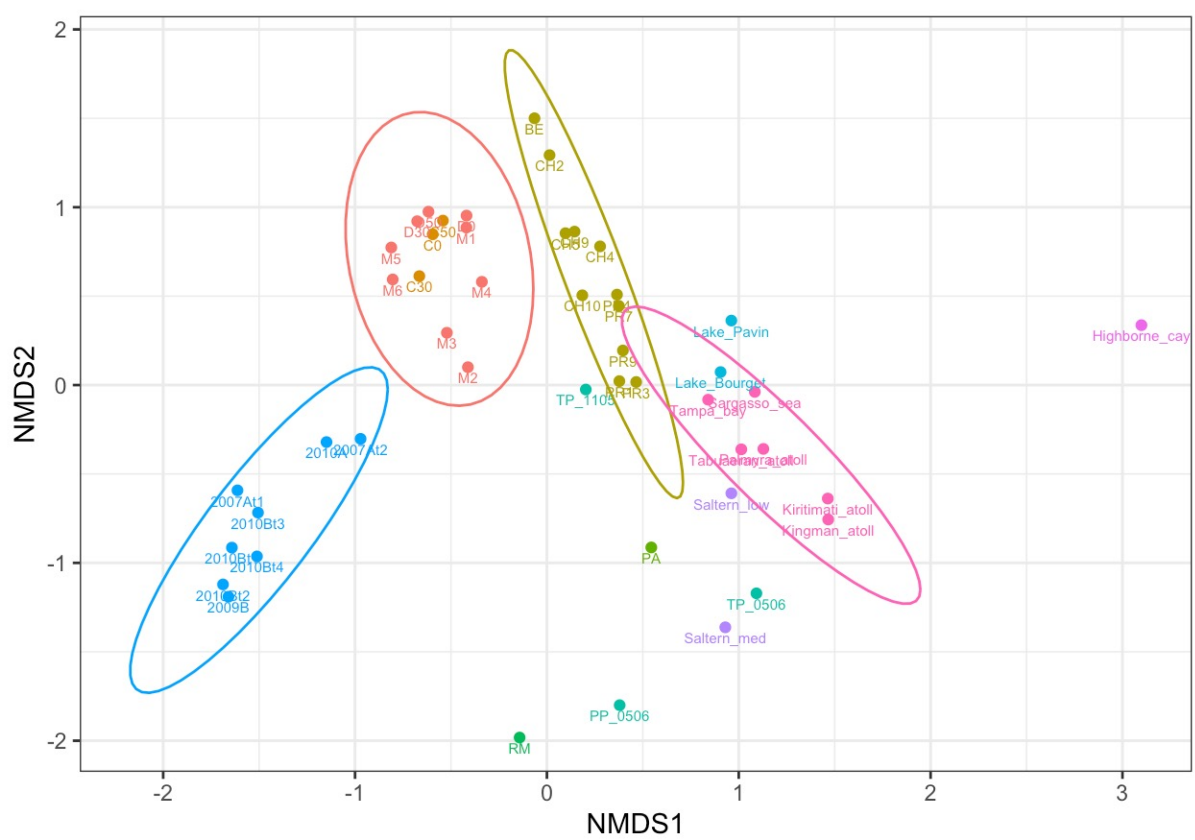

Supplemental Figure 5 NMDS analysis of Bray-Curtis dissimilarities among 47 viromes. ellipses represent 95% confidence interval for a multivariate t distribution. AD viromes are represented by red points agglomerated inside a red ellipse. Other CCB viromes (PR and CH) are represented by olive green points aggregated inside an olive green ellipse. Sea viromes are represented by pink points inside a pink ellipse. High hypersaline viromes are represented by blue points inside a blue ellipse.

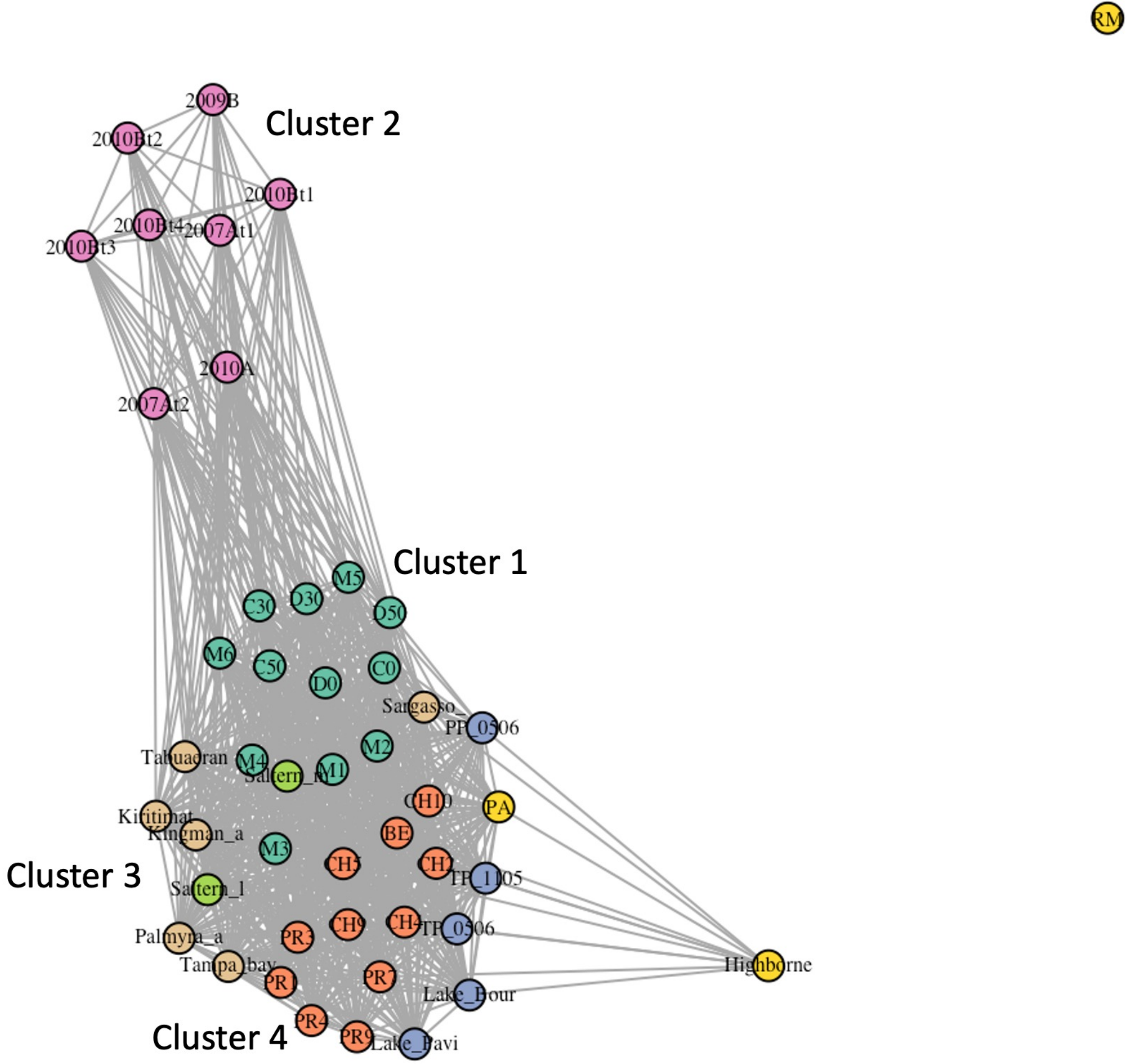

Supplemental Figure 6 Bray-Curtis similarity network showing 75% (above 1st quartile) of the strongest similarities. AD late surface and deep viromes are closer to high salinity hypersaline viromes. AD viromes are represented by jade green circles within cluster 1. Other CCB viromes (PR and CH) are represented by orange circles within cluster 4. Ocean viromes are represented by beige circles within cluster 3. High hypersaline viromes are represented by pink circles within cluster 2.

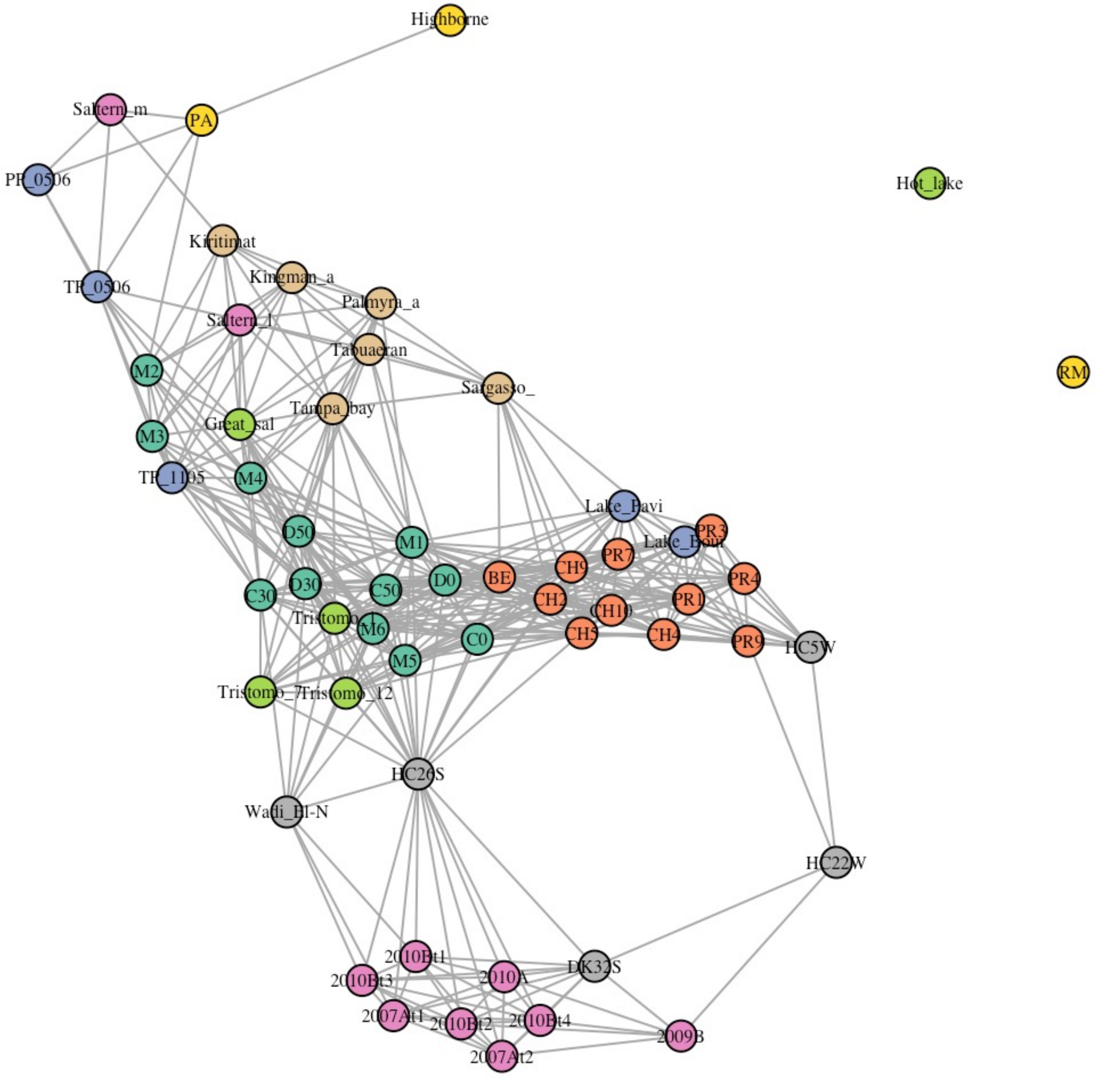

Supplemental Figure 7 OTU level Bray-Curtis similarity network showing 25% (above 3rd quartile) of the strongest similarities. AD viromes are represented by jade green circles. Other hypersaline microbial mats are represented by lime green circles. Soda lakes viromes are represented by grey circles. Other CCB viromes (PR and CH) are represented by orange circles. Ocean viromes are represented by beige circles. High hypersaline viromes are represented by pink circles within.

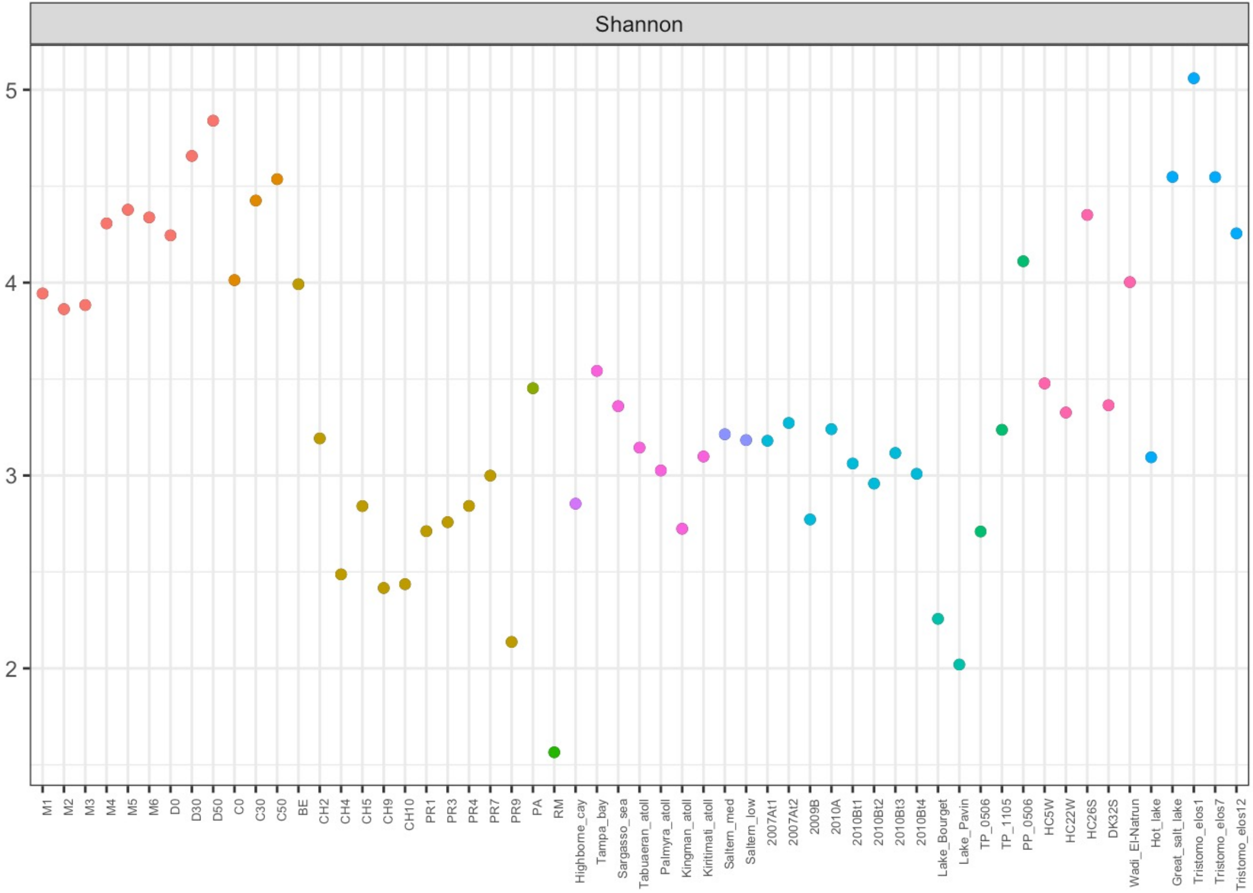

Supplemental Figure 8 OTU level alpha diversity index (Shannon) including five viromes from hypersaline microbial mats (blue points) and soda lakes (magenta points), respectively.
